# Investigation of the Influence of Thermodynamic and Kinetic Flexibility of Polymer Chains in Thermoplastic Polyimides on Their Thermal and Mechanical Properties: Experiment and All-Atom Computer Simulations

**DOI:** 10.3390/polym18131624

**Published:** 2026-06-30

**Authors:** Victor M. Nazarychev, Natalia V. Lukasheva, Andrei L. Didenko, Vera E. Sitnikova, Ivan V. Abalov, Vladislav V. Kudryavtsev

**Affiliations:** 1 Branch of Petersburg Nuclear Physics Institute Named by B.P. Konstantinov of the National Research Centre «Kurchatov Institute»—Institute of Macromolecular Compounds, St. Petersburg 199004, Russia; 2 The Center for Chemical Engineering, ITMO University, Saint Petersburg 197101, Russia

**Keywords:** polyimides, molecular dynamics simulations, glass transition temperature, persistence length, local orientational mobility, mechanical properties

## Abstract

The impact of force field models on the thermal and mechanical characteristics of polyimides was comprehensively examined for the first time. Polyimides (PI) are heterocyclic polymers with outstanding thermal and chemical stabilities and excellent dielectric properties. In this study, we used all-atom molecular dynamics (MD) simulations to examine how the flexibility of the dianhydride fragment affects the thermal and mechanical properties of three polyimides: PMDA-ODA, ODPA-ODA, and R-ODA. The considered polyimides have different dianhydride fragments based on pyromellitic acid (PMDA), tetracarboxylic acid diphenyl oxide (ODPA) and 1,3-bis(3′,4-dicarboxyphenoxy)benzene acid (R), with a constant diamine: 4,4′-oxydianiline (ODA). Models were built using five classical force fields (OPLS-AA, Amber/GAFF, Gromos, Charmm/CGenFF, and UFF). For each polyimide, eight models were generated using different force fields and charge schemes: (i) OPLS-AA with 1.14*CM1A charges, (ii) OPLS-AA with HF/6-31G* (RESP) charges, (iii) GAFF with AM1-BCC charges, (iv) GAFF with HF/6-31G* (RESP) charges, (v) CGenFF (version 4.6) with native charges, (vi) CGenFF (version 5.0) with native charges, (vii) Gromos54a7 with native charges, and (viii) UFF with QEq charges. The difference in the chemical structures of the polyimide repeating unit leads to differences in the thermodynamic and kinetic flexibilities that affect the thermal and mechanical properties. Simulations of glass transition temperatures (*T_g_*) for three polyimides PMDA-ODA, ODPA-ODA, and R-ODA mostly replicate the experimental order *T_g_*(PMDA-ODA) > *T_g_*(ODPA-ODA) > *T_g_*(R-ODA), except for the CGenFF (version 4.6) force field. The experimental density ratio *ρ*(PMDA-ODA) > *ρ*(ODPA-ODA) > *ρ*(R-ODA) is most accurately replicated by OPLS-AA (RESP) and CGenFF (version 5.0) polyimide models. The coefficients of thermal expansion (CTE) correspond with the experimental trend, exhibiting an increase in the following order: PMDA-ODA < ODPA-ODA < R-ODA. Gromos54a7 precisely delineates both the ratio and absolute values CTE for all polymers. OPLS-AA (RESP), OPLS-AA (CM1A), CGenFF (version 4.6), and UFF (QEq) models replicate PMDA-ODA’s CTE, while GAFF (RESP) and GAFF (AM1-BCC) models replicate ODPA-ODA and R-ODA CTE values. The ratio between the simulated values of Young’s modulus, yield strength, and strain-hardening modulus followed the sequence PMDA-ODA > ODPA-ODA > R-ODA for the OPLS-AA (RESP) and CGenFF (version 5.0) models.

## 1. Introduction

Polyimides (PIs) are heterocyclic polymers with high thermal and chemical resistance and good dielectric properties [1,2,3]. Among these polymers, the commercial polyimide Kapton^®^ produced by the DuPont company is a well-known heat-resistant polymer that is widely used for production polymer films in the electrical and electronic engineering [4,5]. Kapton^®^ polyimide is characterized by its high viscosity owing to the relatively rigid structure of its repeating unit. In turn, a modification of the chemical structure of Kapton^®^ polyimide could also be proposed [6,7,8], or a search for a replacement for this commercial product using polymers with improved properties available on the market could be conducted [6,7].

This has prompted research into the discovery and development of new polyimides with analogous properties but featuring a more flexible chemical structure of the repeating unit. This allows these polymers to be processed from the melt at lower temperatures without the risk of thermal degradation. Even a small change in the chemical structure of polyimides leads to a significant change in their performance properties, allowing them to possess thermoplastic properties [9,10]. It was observed for polyimides with differences only in the structure of the dianhydride fragment of the polyimide repeating unit [11,12].

**Figure 1:**
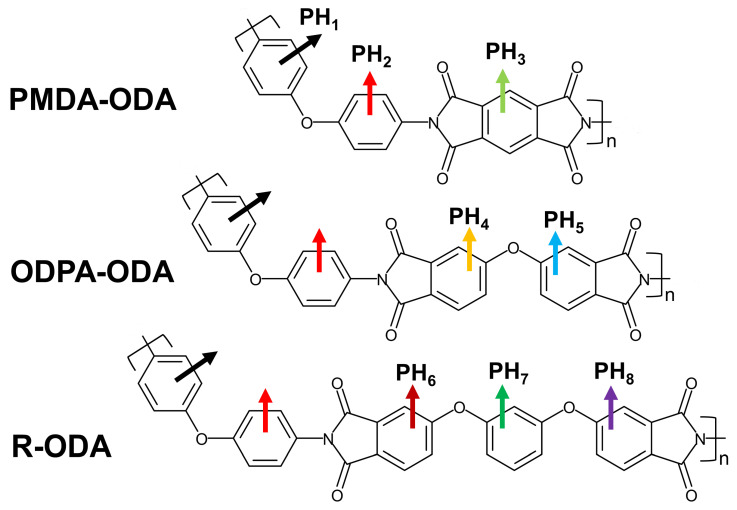
Chemical structures of polyimide PMDA-ODA, ODPA-ODA, and R-ODA repeating units. The arrows indicate the normal vectors to the planes of the PH_1_–PH_8_ rings of phenylene and phthalimide; their local orientational mobilities were studied in this study.

Experimental studies have shown that increasing the flexibility of the polymer chain can significantly affect the thermal stability, strength, and ultimate deformation of polymers [13,14]. In this study, together with PMDA-ODA, two other polyimides, ODPA-ODA and R-ODA (Figure 1), which have the same diamine as in the chemical structure PMDA-ODA and more flexible ODPA and R dianhydrides, were considered. In our previous work [15], we conducted an experimental study to examine the impact of several key factors, including the choice of solvent and imidization temperature of the prepolymer, on the alteration of the thermal and mechanical properties of the polyimides, employing a range of experimental techniques. The present study continues our experimental measurements of the properties of Kapton-like polyimides with the aim of establishing a structure–properties relationship.

By comparing the experimental data of the thermal and mechanical properties of polyimide Kapton^®^ with those of polyimides with different chemical structures in the dianhydride fragment, the molecular mechanisms (change in the chain flexibility) responsible for improving the final performance properties of these polyimides can be supposed [16,17]. The main problem is that, to date, experimental methods for studying polyimide materials alone cannot allow us to unambiguously establish the molecular mechanisms responsible for changing the thermal or mechanical properties of polymers. Therefore, extensive computer studies were conducted to select the most suitable computer all-atom model to accurately replicate the thermal and mechanical properties of these polymers.

All-atom computer simulations allow the investigation of the intra- and intermolecular interactions of polymers to determine the molecular mechanisms underlying the changes in their performance properties [18,19,20]. To perform all-atom computer simulations to obtain a correspondence between experimental and simulation results, it is necessary to correctly select the intra- and intermolecular interaction potentials of polyimides [21,22]. The importance of a correct choice of interaction potentials was demonstrated by comparing the results of experimental and computer simulation studies of Kapton^®^ polyimide [4,5]. For new polyimides studied only experimentally, the selection of the interaction potentials that correctly describe the experimental results will be investigated in the present study.

A direct comparison of computer simulation and experimental results is necessary to select the correct model parameters to reproduce the experimental polymer characteristics in the simulation. It would be ideal to have one force field for an all-atom computer simulation that covers a wide range of performance characteristics, and for this force field to give quantitative agreement between the experimental and simulation characteristics of the polymer systems under study.

It should be noted that developing such a unique singular force field is rather difficult. Typically, for computer simulations, the developed models are mainly used to predict only one type of the performance properties. Regarding reaching quantitative agreement between the simulated and experimental data for thermophysical and mechanical characteristics they can only be compared qualitatively because of the many-fold difference in cooling and deformation rates [19,23] and as well as small system sizes and short timescales (nanoseconds to microseconds) in the simulations, which do not allow the experimental long-time relaxation process to be captured. For these reasons the simulated values strongly differ from the experimental data. Usually to overcome this problem the extrapolation procedure is used [19,24], but this way is very time-consuming and can be successfully applied, for example, for one polymer system studied by using a number of force fields.

When a series of polymers is studied and a large number of force fields is applied a different approach can be used. Although the simulation values strongly differ from the experimental values, they follow the same trend as the experimental data in a series of polymers studied, showing which material has higher or lower characteristics [9,21].

For polyimides, a systematic investigation of the existing polymer force fields for the prediction of both thermal and mechanical properties remains a complex task. In the literature, only a few studies have compared the force fields for one type of polyimide properties prediction. In particular, Odegard et al. [25] compared the OPLS-AA, Amber, and MM3 force fields for the description of the mechanical properties of polyimide BPDA-API. Previously our investigation [26] showed that the Gromos and Amber force fields might be used for the prediction of the thermal properties of thermoplastic polyimides ULTEM^TM^ and EXTEM^TM^. However, Kapton-like polyimides have mainly been simulated using one force field for the prediction of mechanical (OPLS-AA) [27], thermal (Compass, PCFF+) [28,29], thermal-degradative (ReaxFF) [30] and water-transport (Tripos) [31] properties. In recent years, polymer simulations using machine-learning interatomic potentials (MLIPs) have shown promise, enabling more flexibility and near-quantum mechanical precision at lower processing costs than ab initio molecular dynamics [32]. Crystalline polymer simulation, oligomer and polymer chain dynamics, and polymer glass transition prediction were confirmed using these methods. To deploy MLIPs, high-quality quantum-mechanical training data and thorough confirmation of their transferability to specific polymer chemistries and topologies are required. Simulating complex systems, longer trajectories, and direct comparisons with earlier polymer simulation studies is easier using a classical force-field molecular dynamics framework.

This study offers the first thorough comparison of all-atom models in molecular dynamics simulations of complicated heterocyclic polymer-like polyimides. These polyimides have chemical structures with aromatic rings that may exhibit substantial intermolecular interactions. In the current research the attention is focused on the applicability of the most general classical force fields for Kapton and Kapton-like polyimides that differ by their chain flexibility due to the differences in the dianhydride fragments. The studied three heat-resistant polyimides based on the radical residues of diamine 4,4‘-diaminodiphenyloxide (ODA) and three dianhydrides: benzene-1,2,4,5-tetracarboxylic dianhydride (PMDA), 4,4′-oxydiphthalic anhydride (ODPA), and dianhydride 1,3-bis-(3′,4-dicarboxyphenoxy)benzene (R), as shown in Figure 1.

For these polyimides, models based on the five most common classical force fields (OPLS-AA [33,34], Amber [35], Gromos [36], Charmm [37], and universal force field (UFF) [38]) were developed. Eight distinct models for each polyimide were developed by employing various force fields and atomic partial charges calculation methods: 1. OPLS-AA force field with CM1A [39] partial charges; 2. OPLS-AA force field with HF/6-31G* (RESP) partial charges; 3. GAFF [40,41] force field with AM1-BCC [42] partial charges; 4. GAFF force field with HF/6-31G* (RESP) partial charges; CGenFF force field [43] with native charges for two versions: 5. CGenFF version 4.6 [44]; 6. CGenFF version 5.0 [45]; 7. Gromos54a7 force field [46] with predefined partial charges assigned to atoms within the force field’s parameter sets; and 8. UFF force field with QEq [47] partial charges was used. Thus, 24 systems were simulated and thermodynamic and mechanic characteristics were calculated for each system. The effects of different force fields on the calculated base thermal and mechanical characteristics were examined together with the detection of these properties’ correlation with the calculated chain flexibilities. The criterion for applicability of the considered models was mainly the reproduction in the calculated characteristics of the experimentally obtained trend for the three polyimides. The only characteristic that can be considered as convenient for comparison with experimental values is the coefficient of thermal expansion (CTE), which as it was showed in our previous study [19] is practically independent of the thermal history of the sample.

## 2. Materials and Methods

### 2.1. Materials

Most of the experimental values used in this study were obtained as is or recalculated from the data presented in Ref. [15]. To determine the value of the linear thermal expansion coefficient, additional synthesis of thermoplastic polyimide samples was performed, and the values were measured.

The subsequent reagents served as initial monomers for the synthesis of the examined PIs: benzene-1,2,4,5-tetracarboxylic dianhydride (PMDA) (Sigma-Aldrich, Saint Louis, MO 63103, USA, 99%, CAS#: 89-32-7, *T_m_* ~ 283–286 °C (lit.)); 3,3′,4,4′-oxydiphthalic anhydride (ODPA) (Sigma-Aldrich, Saint Louis, MO 63103, USA, 98%, CAS#: 1823-59-2, *T_m_* ~ 225–229 °C (lit.)); 4,4′-oxydianiline (ODA) (Tokyo Chemical Industry, Tokyo, Japan, 98%, CAS#: 101-80-4, *T_m_* ~ 188–192 °C (lit.)); dianhydride 1,3-bis(3′,4-dicarboxyphenoxy)benzene (R) (TechKhimProm LLC, Yaroslavl, Russia, *T_m_* ~ 164 °C). The solvents N,N-dimethylacetamide (DMAc) (J-SC Vecton, Sant-Petersburg, Russia, Chemical Grade), N,N-dimethylformamide (DMF) (J-SC Vecton, Sant-Petersburg, Russia, Chemical Grade), and N-methyl-2-pyrrolidone (NMP) (J-SC Vecton, Sant-Petersburg, Russia, Chemical Grade) were dehydrated using calcium hydride and subjected to two distillations under an argon atmosphere.

### 2.2. Chemical Synthesis of Polyimides Samples

The synthesis of prepolymers in the form of polyamic acids (PAA) was carried out according to methods described in the literature [1,2,3,48,49,50].

The monomer in an amount of 5.0 g (25 mmol) of 4,4′-oxydianiline was placed in a three-neck flask equipped with a mechanical stirrer and a Drexler nozzle for the argon inlet and outlet. After dissolving the diamine in a solvent (DMAc, DMF, or NMP) at 15 °C, one of the three dianhydrides (PMDA, ODPA, or R) was added in an amount of 25 mmol. After six hours of vigorous stirring at 15 °C, nine polyamic acid (PAA) samples, specifically PMDA-ODA, ODPA-ODA, and R-ODA each in the three amide solvents employed, were synthesized. These samples exhibited weight concentrations ranging from 15% to 20%, which was selected to optimize the molecular weight of the PAA samples. Subsequently, the samples were filtered through a Nutsche filter at a pressure of 10 atm using a metal filtration membrane with a pore size of 10 µm. This process was intended to remove low molecular weight gel agglomerates, which are reaction by-products, and the samples were then degassed under vacuum conditions. Extended information on the chemical synthesis of the considered polyimides is available in [15].

### 2.3. Experimental Techniques

The specifics of the measurements performed via thermal mechanical analysis (TMA and DTMA), dynamic mechanical analysis (DMA), density measurements, and mechanical property calculations can be found in our previous study [15].

The linear coefficient of thermal expansion (CTE) of the film materials in the temperature ranges of 25–180 °C (ODPA-ODA and R-ODA) and 25–200 °C (PMDA-ODA) were determined in the tensile mode using a TMA 402 F1 thermomechanical analyzer (NETZSCH, Selb, Germany). A stress of 5 kPa was applied to the samples. The heating rate was 2 °C/min. The thickness, width, and length of the samples were 50 μm, 4 mm, and 10 mm, respectively.

### 2.4. Computer Models and Simulation Techniques

#### 2.4.1. Force Fields

The flexibility characteristics of the studied polyimides obtained using different force fields may have different values. It is worth noting that different force fields can consider or reproduce this flexibility in different ways, which can subsequently lead to differences in performance properties. Therefore, the focus of this work was changed to describe the intra- and intermolecular interactions in computer simulations of these polymers using the most commonly applied force fields belonging to different families, such as Amber, OPLS-AA, Charmm, and Gromos. The largest number of atom types in the Amber family of force fields is embedded in the GAFF force field. In this force field, the parameterization of electrostatic interactions is performed by calculating the atomic partial charge values using two quantum-chemical methods: the semi-empirical AM1-BCC and the non-empirical HF/6-31G* (RESP).

In the case of the OPLS-AA force field the partial charges were calculated using the 1.14*CM1A calculation method, which is the standard for this force field. Also, partial charge values were calculated using the HF/6-31G* (RESP) calculation method, which as the results of other works have shown can be used in the computer simulation of individual polymers. In the Charmm General Force Field (CGenFF) and Gromos54a7 force fields, predefined partial charges assigned to atoms within the parameter sets of the force field were used. We evaluated two iterations of the CGenFF force field, specifically versions 4.6 and 5.0. These versions were integrated into two online platforms designed to generate topology and coordinate files for Charmm force fields. For CGenFF version 4.6 (CGenFF_v.4.6), all interaction parameters were sourced from the “Ligand Reader & Modeler” module [44] of the CHARMM-GUI web server [51]. In contrast, for CGenFF version 5.0 (CGenFF_v.5.0), the interaction parameters were obtained from the web server cgenff.com. The CGenFF_v.5.0 is the latest CGenFF force field version and its comparison with a previous version might be important for the final property’s prediction of the studied polymers. Gromos54a7 is the one of the latest representatives of the Gromos family of force fields available through the ATB online server. In addition, computer simulations of materials containing both polymer binders and metal or metal oxide particles using the atomic UFF force field are common in the literature. It should be noted that to account for electrostatic interactions in this force field, the values of partial charges are calculated using the semi-empirical method QEq. All considered models have its own functional form of potential functions [52], as shown in Paragraph S1 and Table S1 in the Supplementary Materials.

Although the PCFF has been successfully applied to various polymer systems [29,53], it was not included in the present study due to the use of Gromacs, which does not offer a comprehensive and validated implementation of PCFF as a standard force-field package. As a class-II force field, PCFF necessitates additional bonded cross-terms and non-bonded treatments beyond those available in the Gromacs-compatible force fields selected for this research. The conversion of PCFF parameters could introduce uncertainties and would require independent verification. This study concentrates on the CGenFF, GAFF, OPLS-AA, UFF, and Gromos54a7 force fields, which are compatible with the Gromacs and offer established parameterization methodologies for the studied polyimides. Previously, all considered force fields, except CGenFF, have been employed in the computer simulation of polyimides [18,22,25]. Therefore, this study primarily focuses on the general and widely used force fields that are extensively applicable to computer simulations of polymers.

#### 2.4.2. Creating of Initial Samples in Computer Simulations

Comparing the thermomechanical properties of polyimides with different chemical structures is a difficult methodological task from the perspective of all-atom computer simulations. The initial development of polyimide samples, utilizing all eight selected models for each polyimide type, is a computationally demanding process. To standardize these samples prior to analyzing their thermophysical and mechanical properties, they were compressed and equilibrated using a model based on the OPLS-AA_CM1A model. At a temperature of 1200 K, the values of the average distance between the ends, the radius of gyration, and the characteristic ratio were calculated for all models considered, as shown in Figure S1 in the Supplementary Materials. Consistency was maintained for the atom names across all eight models. Thus, this helped to make the transition between models straightforward during the process of generating samples in the equilibrium state for all considered models. Computer simulations were performed using the atomistic molecular dynamics package Gromacs (v. 2022) [54].

An analysis of the scientific literature [55] showed that in the computer simulation of PMDA-ODA, the minimum degree of polymerization at which the glass transition temperature value starts to depend weakly on the molecular weight of the polymer (known as the polymer regime) is equal to 12. The degrees of polymerization of the other two polyimides, ODPA-ODA and R-ODA, were chosen in analogy with that of PMDA-ODA. Thus, the total number of atoms in the polymer chains of PMDA-ODA, ODPA-ODA, and R-ODA were 470, 602, and 734, respectively. Initially, 27 partially coiled polymer chains were randomly placed in a periodic box of 50 nm on each side [18]. The initial stage of the computer simulations was performed without considering the electrostatic interactions. The partial charges are assumed to be zero. To create block samples, multistage compression was used at a temperature of 800 K and a pressure of 50 to 300 bar, followed by a multistage reduction in pressure to 1 bar [18]. During this process, constant temperature and pressure values were maintained by applying a Berendsen thermostat and barostat, for which the relaxation times *τ_t_* and *τ_p_* were equal to 0.1 ps and 1 ps, respectively. After compression, 1.5 µs equilibration simulations were performed at 800 K, during which the sizes of the polymer chains reached their limiting values (Figure S1 in the Supplementary Materials). A time step of 0.5 fs was used in the compression simulations. During the equilibration, cooling, and deformation phases, the simulation time step was set to 1 fs for all systems. For most of the models analyzed, a 1 fs simulation step guaranteed stability in the simulations. Models using the Gromos54a7 force field demonstrated improved stability at temperatures over 800 K with a simulation time step of 0.5 fs.

It is noteworthy that distinct cut-off radii for the Lennard–Jones and Coulomb potentials were selected for each force field. The cut-off radii for van der Waals and electrostatic interactions, as well as the thermostats and barostats employed for the CGenFF, OPLS-AA, GAFF, and Gromos54a7 force fields, were consistent with the parameters utilized in our previous studies [56] and based on the original parameterization of each force field. Namely, the van der Waals interactions were limited to a specific distance for each force field *r_vdw_*, with the following values: 0.9 nm for GAFF, 1.2 nm for CGenFF, 1.3 nm for OPLS-AA, and 1.4 nm for Gromos54a7. For the UFF force field, a cut-off radius of 1.2 nm was applied to the Lennard–Jones. Additionally, in the CGenFF and OPLS-AA force fields, a switching function is used to adjust the van der Waals interactions. The CGenFF force field uses a function that gradually decreases the forces to zero within the range from 1.0 nm to *r_vdw_* − 1.2 nm. In contrast, the OPLS-AA force field smoothly reduced the interaction energy to zero between 1.1 nm and *r_vdw_* − 1.3 nm. In line with the original parameterizations of the force fields considered, beyond the cutoff distance, either an energy correction (GAFF) or corrections to both the energy and pressure (OPLS-AA and UFF) are applied. For the electrostatic interactions in the models, the cutoff radius for the direct space summation was set to *r_vdw_*. The neighbor list was refreshed every 10 steps for all models. For a more accurate computer simulation of polyimide in every considered all-atom model, systematic variation in the cut-off radius might help to enhance the tuning of the experimental property prediction in computer simulation. However, in the current study, to eliminate the extensive validation of the bonded and non-bonded parameters, we used the default values of the simulation characteristics for each force field.

A V-rescale (OPLS-AA, Gromos, and UFF force fields) or Nose-Hoover (CGenFF, and GAFF force fields) thermostat and a Parrinello-Rahman barostat, with relaxation times *τ_t_* and *τ_p_* set to 0.1 ps and 2 ps, respectively, were employed to maintain constant temperature and pressure in the computer simulations.

The values of the partial charges were then instantaneously turned on, and additional computer simulations were performed for 500 ns at 1200 K. In this case, the temperature was instantly changed from 800 to 1200 K. For each sample for the selected type of force field model, computer simulations were performed at 1200 K from 100 to 700 ns, during which the polymer chains for all selected models moved on their average polymer sizes (Figures S2 and S3 in the Supplementary Materials). The three initial configurations of the polymer samples in different models were created by selecting the configurations of the polymer that were separated in time by an interval longer than the time required for the polymer chains to move on their average sizes. Thus, the initial three configurations of all the considered PIs were created for each of the seven chosen models.

The whole time of the simulations, including compression, equilibration, cooling, and deformation of the samples, was around 35 μs. Therefore, doing comprehensive molecular dynamics simulations would be almost impracticable without using the Gromacs tool. The comprehensive performance of each model assessed for each polyimide is shown in Table S2 in the Supplementary Materials. The main simulations were performed using AMD EPYC 9654 processor with 96 physical cores and 192 logical processors, together with two GeForce RTX 4080 SUPER Phoenix GS 16GB GPUs (Gainward, Shenzhen, China). Depending on the polymer and force field model, the measured throughput ranged from 415 to 448 ns/day for PMDA-ODA, from 364 to 408 ns/day for ODPA-ODA, and from 328 to 372 ns/day for R-ODA. The findings in Table S2 in the Supplementary Materials demonstrate that the models based on the Gromos54a7 and UFF force fields had slightly better performance among all the polyimides.

## 3. Results and Discussion

### 3.1. Thermal Properties

#### 3.1.1. The Glass Transition Temperature from Simulations and Experiment

The glass transition temperatures were determined from simulations using two approaches: density–temperature relationships and analyses of the translational mobility of polymer chains (see below in Section Value of *T_g_* Calculated from Density–Temperature Relationships and Section Value of *T_g_* from Mean Squared Displacements, respectively). Comparable discrepancies between glass transition temperatures determined from density–temperature relationships and those inferred from analyses of the translational mobility of polymer chains have been reported in both investigations of thermal properties and other related studies [57,58]. The values of the glass transition temperatures determined from the simulations are compared with the experimental ones which were taken as the averages for each polyimide according to the solvent type used for synthesis and the experimental method used to measure the glass transition temperature (see below in the Section Comparison of the Calculated and the Experimental *T_g_*). In simulations, glass transition temperatures can only be compared qualitatively with experimentally determined values *T_g_*. The discrepancy commonly observed between the simulated and experimental glass transition temperatures can be attributed to the approximately ninefold difference in cooling rates [19,23]. The simulated values of *T_g_* are usually much higher than the experimental values, but they must follow the same trend as the experimental data, showing which material has a higher or lower *T_g_* [9,21].

##### Value of *T_g_* Calculated from Density–Temperature Relationships

**Figure 2:**
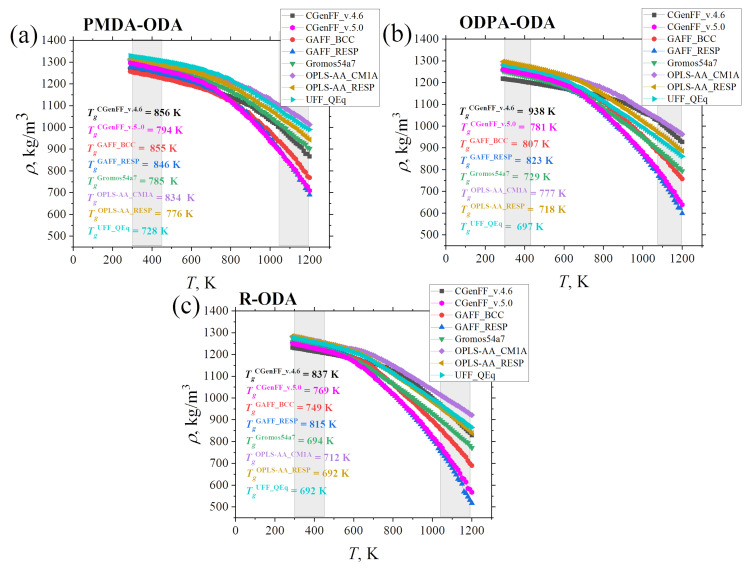
Average temperature dependences of the density of three polyimides: (**a**) PMDA-ODA, (**b**) ODPA-ODA, and (**c**) R-ODA. The gray areas where the curves were approximated are highlighted in the figure. The glass transition temperatures obtained from this approximation are also shown in this figure.

To calculate the glass transition temperature, the densities of the systems during the cooling procedure at a cooling rate of 1.5 × 10^11^ K/min (10 K/4 ns) were determined. The temperature dependence of the density can be approximated by two linear sections corresponding to high (1050–1200 K) and low (300–450 K) temperatures. The glass transition temperature is defined as the temperature at which the intersection of these two linear approximations is observed. The areas for calculating the glass transition temperature were selected using the proposed methodology [18,19]. The temperature dependence of the density averaged over three configurations is presented in Figure 2, and the calculated glass transition temperatures are listed in Table S3 in the Supplementary Materials.

This study employed three additional prevalent methodologies to ascertain the glass transition temperature by investigating the temperature-dependent density. The findings indicate that the glass transition temperatures derived from several methodologies (standard [18,19], Hyperbola [59], and Shapiro methods [60]), which utilize distinct strategies (the description of these strategies is given in [61,62,63]) for selecting linear segments for data approximation, produce comparable results for each polyimide model examined (Figure S4 in the Supplementary Materials).

Analysis of the results showed that all selected methods for calculating the glass transition temperature from the temperature dependence of the density demonstrate a qualitatively identical relationship between the glass transition temperature values of the studied polyimides. Nevertheless, the optimal ranges for calculating the glass transition temperature for each polymer model differed slightly. In this regard, in order to standardize the calculations of the selected polymers using different models, it was decided in this work to use a method that provides a fixed temperature range (see Figure 2) for determining the glass transition temperature of the studied polyimides.

The temperature dependence of the density for the different polyimide models exhibits substantial variation across the examined temperature range. In particular, for the GAFF_RESP, CGenFF_v.5.0, GAFF_BCC, and Gromos54a7 models, the difference between the density values in the molten (*T* = 1200 K) and glassy (*T* = 290 K) states is more pronounced. This enhanced density contrast is presumably attributable to weaker intermolecular interactions among the polymer atomic structures described by these models. Analysis of the temperature dependencies of the density for each polyimide shows that, at high temperatures, the models considered demonstrate different density values. The density values of all three polyimides increase in the order of GAFF_RESP, GAFF_BCC, Gromos54a7, OPLS-AA_RESP, UFF_QEq, CGenFF_v.5.0, and OPLS-AA_CM1A models, with the exception of the CGenFF_v.4.6 model, which has a higher density at high temperatures for polyimide ODPA-ODA compared to the density values of polyimides PMDA-ODA and R-ODA.

##### Value of *T_g_* from Mean Squared Displacements

As part of this study, the glass transition temperature was calculated using an alternative method [64] based on the analysis of the dependence of the mean squared displacement (MSD) of the centers of mass of individual polymer chains on the inverse temperature (Figure S5 in the Supplementary Materials). The *T_g_* values obtained are presented in Figure S4 in the Supplementary Materials, together with the values obtained from the densities.

The relationship obtained for the glass temperatures shows the same trend as that calculated from the density–temperature dependence, which is consistent with the experimental ratio of the polyimide glass transition temperatures. The glass transition temperature values obtained from MSD are lower than those obtained using the method based on the temperature dependence of the density. This may be due to the consideration of different scales, which are involved in the process of changing the density of the system and the mobility of individual polymer chains when the temperature is changed.

##### Comparison of the Calculated and the Experimental *T_g_*

**Figure 3:**
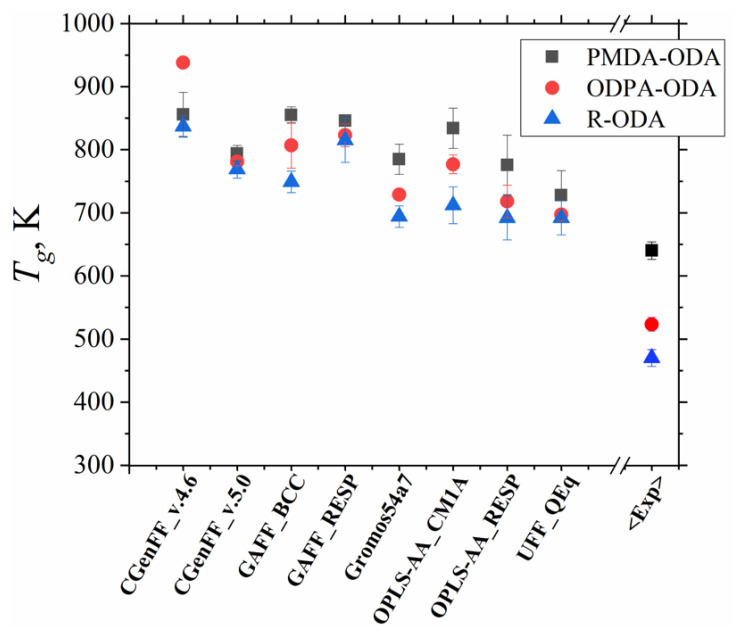
Comparison of the glass transition temperatures obtained from the simulation and experiment. The error value is calculated as the root mean square deviation from the mean value, based on three samples.

The relationship between the glass transition temperatures varies depending on the chosen model, and comparison of the calculated glass transition temperatures with the average experimental values are shown in Figure 3. Previously, the glass transition temperatures of these polyimides, which were synthesized in different solvents, were measured using several experimental methods, and they differed slightly between the polyimides [15]. This allowed us to average the experimental glass transition temperatures for each polyimide according to the solvent type used for synthesis and the experimental method used to measure the glass transition temperature. The experimental *T_g_* values are presented in Figure S6 in the Supplementary Materials.

Previous experimental data showed that the glass transition temperatures of PMDA-ODA, ODPA-ODA, and R-ODA polyimides have the following ratio: $T_{g}^{PMDA-ODA}\;>\;T_{g}^{ODPA-ODA}\;>\;T_{g}^{R-ODA}$ [15].

The data obtained from our simulations confirmed that the relationship between the glass transition temperatures obtained experimentally was qualitatively reproduced for all models, except for CGenFF_v.4.6. In addition, although the ratio between the glass transition temperatures (obtained experimentally) is preserved for the CGenFF_v.5.0 and GAFF_RESP models, their values are very similar. To find out the reason of the excess of the *T_g_* for the ODPA-ODA over PMDA-ODA indicated for the CGenFF_v.4.6 model, the centroid RDF, where the centroid is the arithmetic mean of the positions of the aromatic rings six carbons, was calculated (Paragraph S2, Equations (S1)–(S3), Figures S7–S10 in the Supplementary Materials) at high temperature (in melt). The functions constructed for ODPA-ODA revealed for the CGenFF_v.4.6 the existence of the maximum at the distance ~ 0.35 nm corresponding to the stacking interactions [65] in contrast to the other models (Figure S7 in the Supplementary Materials). The additional search of the origin of these interactions analyzing the parameters of the force field showed that the atomic partial charges in cyclic fragments in the CGenFF_v.4.6 have higher values than in the other models (Figure S11 in the Supplementary Materials). The presence of an atomic oxygen hinge in the dianhydride fragment allows the phthalimide rings of adjacent chains to adjust to each other to implement stacking. The absence of the stacking maxima (Figures S8 and S9 in the Supplementary Materials) for PMDA-ODA and R-ODA can be explained by the features of their chemical structures, which do not allow fragments packing in phenyl rings on both sides of the pyrromellitimide cycle for PMDA-ODA preferring to be in a propeller conformation with the pyrromellitimide ring and the phenyl ring in the meta-connection in the hinge group for R-ODA. The simulations with the charge scaling (0.8) give for ODPA-ODA the results without maximum at the stacking distance, and this result is confirmed by the CGenFF_v.5.0 model for which the atomic partial charges have lower values comparable with the values in the other models (Figure S8 in the Supplementary Materials). Although the reduction in charge values in CGenFF_v.5.0 gives the elimination of the artifact interactions for ODPA-ODA and the correct trend of the *T_g_* values, they are very close. The reduction in the charge values only does not allow us to reproduce the ratio of quantities corresponding to the experiment. As it was mentioned above, very close *T_g_* values for different polyimides were obtained also for the GAFF_RESP model in contrast to the GAFF_BCC model reproducing the experimentally observed differences between values of *T_g_* for the three polyimides. The origin of the wrong result for the GAFF_RESP is the increased (in comparison with the GAFF_BCC) values of the atomic partial charges of the bond dipoles near the hinges resulting in an additionally hindered internal rotation (Figure S11 in the Supplementary Materials).

Value of *T_g_* is dependent on the mobility of the polymer chains which, all other things being equal, is determined by the thermodynamic and kinetic flexibilities of the polymer chains. Thermodynamic flexibility refers to equilibrium state, and kinetic flexibility relates to the rate of the chain segments cooperative rearrangement, which depends on the intramolecular rotation barriers and strength of the intermolecular interactions. Below in the Section 3.1.2 and Section 3.1.3 the calculated values of the thermodynamic and kinetic flexibilities are discussed respectively.

#### 3.1.2. Thermodynamic Flexibility

To evaluate the thermodynamic flexibility of the polymer chain of the simulated polyimides, the persistence length *l_p_* and the Kuhn segment *b* were calculated.

**Figure 4:**
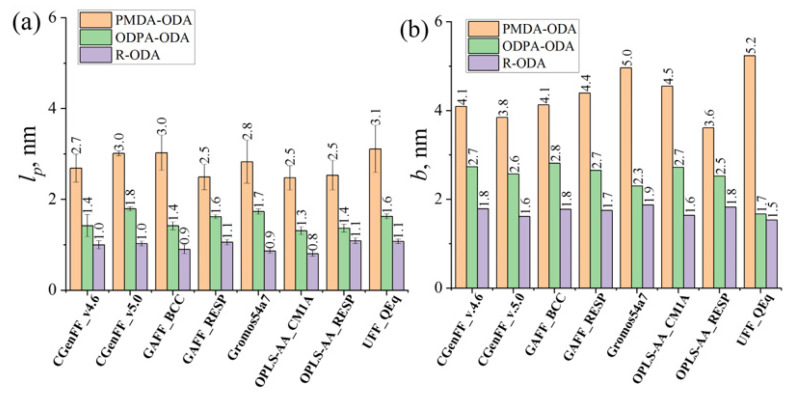
Average values of (**a**) persistence length *l_p_* and (**b**) Kuhn segment *b* of the studied PIs for different models.

The persistence length is a critical determinant of polymer chain flexibility. To assess the differences in flexibility among the polymer chains of the three considered polyimides, the persistence lengths *l_p_* were calculated for all force field models used. For this analysis, the polymer chain was conceptualized as a series of virtual bond vectors extending between adjacent atoms along the chain, thereby delineating the mobile, flexible, and internally rotating segments of the polymer. An illustration of these virtual bond vectors, together with the procedure and formulas used for calculating the persistence length, are provided in the Supplementary Materials in the Paragraph S3 on Figure S12, Equations (S4) and (S5), Table S4, and Figure S13 in the Supplementary Materials. Furthermore, the values of persistence length and Kuhn segments were calculated, as shown in Figure 4.

The ratios for the persistence lengths and the Kuhn segments are reproduced qualitatively for all models. A comparative analysis of the persistence length and Kuhn segment values showed that the flexibility of the polymer chain differs among the polyimides. PMDA-ODA polyimide has the highest persistence length value, ranging from 2.5 to 3.1 nm, depending on the selected model. The presence of a hinge oxygen atom in the ODPA dianhydride of the ODPA-ODA polyimide leads to a decrease in its persistence length by almost 1 nm, reducing its rigidity. Polyimide R-ODA exhibited the greatest thermodynamic flexibility among the three polyimides, with a persistence length ranging from 0.9 to 1.1 nm. The results obtained correlate well with the results of the Kuhn segment calculation. The Kuhn segment values, depending on the selected model, reproduce the ratio observed for the persistence length values: *b^PMDA-ODA^* > *b^ODPA-ODA^* > *b^R-ODA^*, as shown in Figure 4b. It is worth noting that the ratio between the Kuhn segment values and the persistence lengths deviates from the value of two, which is apparently due to simulation conditions not allowing one to reproduce free rotation chain models, as shown in Figure S14 in the Supplementary Materials.

#### 3.1.3. Local Orientational Mobility

The orientational mobility of polymer groups represents the movement of polymer segments and is linked to the value of *T_g_*. The activation energy (*E*_*a*_) is a measure of the energy barrier that must be overcome for this mobility to occur, which means that higher activation energies correspond to less mobility and higher glass transition temperatures. This relationship is critical because it governs the physical properties of the material and its temperature range of use.

The use of the different force field models to describe the intra- and intermolecular interactions of the polyimides under consideration should have a significant effect on the local orientational mobility of individual fragments of the repeating unit of polyimides. Differences in the local orientational mobility of polymer chains determine the differences in their kinetic flexibility. The kinetic flexibility of polymer chains refers to the rate of conformational transformation and mobility of the chain units. This is fundamentally determined by a combination of intramolecular barriers and intermolecular interactions. To compare the differences in the kinetic flexibility of the polymers, the mobility of the units normal to the planes of the aromatic rings (PH_1_–PH_8_) was calculated.

During the cooling step, the local orientational mobility of the phenyl rings’ normal vectors was simulated for both the diamine (PH_1_ and PH_2_) and dianhydride (PH_3_–PH_8_) fragments of the repeating unit, as shown in Figure 1. The first-order Legendre polynomials $P_{1}\left(t\right)$ have been calculated for these vectors [66] (see Paragraph S4 and Equation S6 in the Supplementary Materials). The obtained $P_{1}\left(t\right)$ dependences for PMDA-ODA, ODPA-ODA and R-ODA PIs have been approximated by the Kohlraush–Williams–Watts (KWW) stretched exponentials [66] (see Equations (S7) and (S8) in the Supplementary Materials).

**Figure 5:**
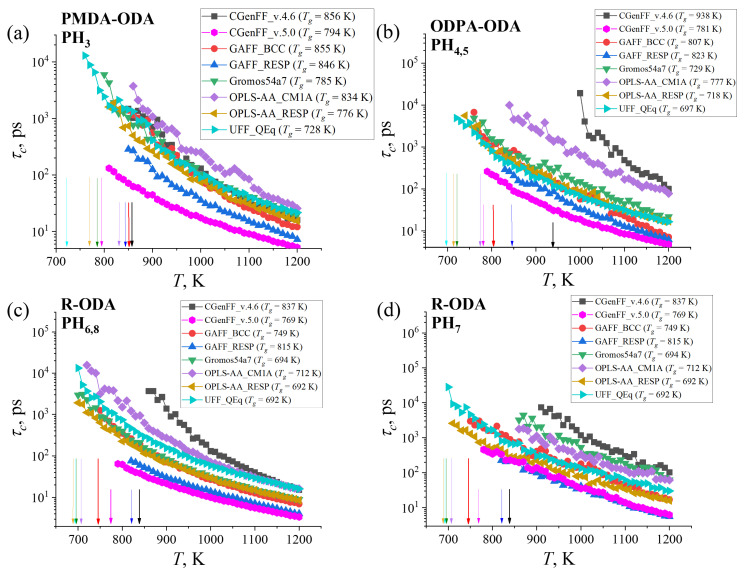
Temperature dependence of the average relaxation times for the orientational mobility of the normal vectors PH_3_, PH_4,5_, PH_6,8_, and PH_7_ for different force field models of the PI samples above the glass transition temperature: (**a**) PMDA-ODA; (**b**) ODPA-ODA; (**c**,**d**) R-ODA. The arrows indicate the glass transition temperatures for different considered models.

To simplify the comparative analysis between the temperature dependencies of the orientational mobility of the normals and the planes of the aromatic rings located in both the diamine and dianhydride fragments of the repeating unit, the relaxation times were averaged: PH_1,2_–PH_1_ and PH_2_, PH_4,5_–PH_4_ and PH_5_, as well as PH_6,8_–PH_6_ and PH_8_. Figure 5 shows the temperature dependencies of the average relaxation time of unit normals to the planes of the aromatic rings located in the dianhydride fragment of the studied polyimides above the glass transition temperature.

The analysis of the obtained data, shown in Figure 5, reveals a strong influence of the all-atom models of the polyimides on the change in the local orientational mobility of the aromatic rings in the dianhydride fragment. In general, the parameters used for the CGenFF_v.5.0 and GAFF_RESP models for all three polyimides considered lead to temperature dependencies that show sufficiently shorter relaxation times compared to the temperature dependencies of relaxation times in other models considered. The orientational mobility of the normal vectors to the phenyl rings located in the diamine fragment of the repeating unit of the polyimides show shorter relaxation time also for the CGenFF_v.5.0 model (Figure S15 in the Supplementary Materials).

Various phenomenological models are used to describe the temperature dependence of relaxation processes in polymer samples of various natures. We used the most commonly used models: Arrhenius [67], mode-coupling theory (MCT) [68,69], and Vogel–Fulcher–Tammann (VFT) [70,71].

At high temperatures, the relaxation time temperature dependencies of all the polyimides considered obey the Arrhenius temperature dependence *τ_c_* ~ A·exp(E_a_/RT); however, as the temperature decreases to the *T_g_* value, a deviation from the Arrhenius dependence is observed, which considers additional contributions from the glass transition process into the local orientation mobility. In this case, the temperature dependence of the mean relaxation time can be well described by the MCT formalism *τ_c_* ~ C·(*T* − *T_c_*)^−γ^ or by the VFT dependence *τ_c_* ~ A·exp(B/(*T* − *T*_0_)), where A, B, and C are approximation parameters, *T*_0_ and *T_c_* are critical temperatures, and *γ* is the mode-coupling theory parameter.

The use of the Arrhenius equation allowed us to determine the activation energies at temperatures ranging from 900 to 1200 K. The results showed that the heterocyclic rings in the dianhydride fragment of the polymer chain (Figure S16a in the Supplementary Materials) exhibited higher activation energies than the activation energies of the planar fragments (phenyl rings) in the diamine fragment for most of the simulation models (Figure S16b in the Supplementary Materials). For the more rigid polyimide PMDA-ODA, the activation energies for both phenylene rings in the diamine fragment are higher than those of the other two polyimides, ODPA-ODA and R-ODA (with the exception of CGenFF_v.4.6, CGenFF_v.5.0 and UFF_QEq), which is consistent with the relationship between the glass transition temperatures (Figure 3) of the three polyimides. The incorrect relationship between *T_g_* (in comparison with the experiment) obtained for the CGenFF_v.4.6 correlates with the relationship between the activation energies of the cyclic fragments in the diamine and dianhydride parts. The weak difference between *T_g_* for the CGenFF_v.5.0 models correlates with the relationship between the activation energies of the phenyl rings in diamine fragments. Reduction in the atomic partial charges in CGenFF_v.5.0 in comparison with CGenFF_v.4.6 leads to a proportional decrease in the activation energies in the dianhydride fragments. For the UFF_QEq the weak differences in the activation energies in diamine and dianhydride fragments correlates with the weak differences in the glass transition temperatures.

The use of the MCT and VFT approaches to describe the relaxation processes in the area near the glass transition allows us to determine the critical temperatures *T_c_* and *T*_0_. Figures S17 and S18 in the Supplementary Materials present the approximation parameters for the temperature dependence of the average relaxation times for the orientational mobility of the unit normal vectors using MCT and VFT. The results show that the critical temperature *T_c_* values for different models differ relative to each other. These temperatures are equal to or slightly higher than the glass transition temperatures of polyimides, calculated from the density–temperature dependence. The *γ* parameter for all systems considered generally ranges from 2 to 4, which is consistent with the results of other studies [24] investigating the dynamics of polymer systems in computer simulations. On the other hand, the critical temperature *T*_0_, determined using the VFT equation is much lower than the glass transition temperature, indicating that processes associated with the local orientational mobility of these heterocyclic fragments occur at lower temperatures, corresponding to the glassy state.

Summarizing the results presented in Section 3.1.2 and Section 3.1.3, it can be concluded that the persistence lengths, which play a key role in determining the glass transition temperature of the polymers for the polyimides considered, are correctly described in all models owing to the close values of the bonded parameters in the corresponding force fields. Polyimide PMDA-ODA, characterized by a high persistence length compared to polyimides ODPA-ODA and R-ODA, which have lower persistence lengths, exhibits a significantly higher glass transition temperature. This relationship between the experimental *T_g_* is not reproduced in the simulations with the CGenFF_v.4.6 model and the strong excess of the *T_g_* for ODPA-ODA over PMDA-ODA is observed. It can be explained by the overestimation of the partial atomic charge values leading to the implementation of strong intermolecular interactions (electrostatic) and to the stacking.

#### 3.1.4. Characteristics in Glassy State

##### Density

**Figure 6:**
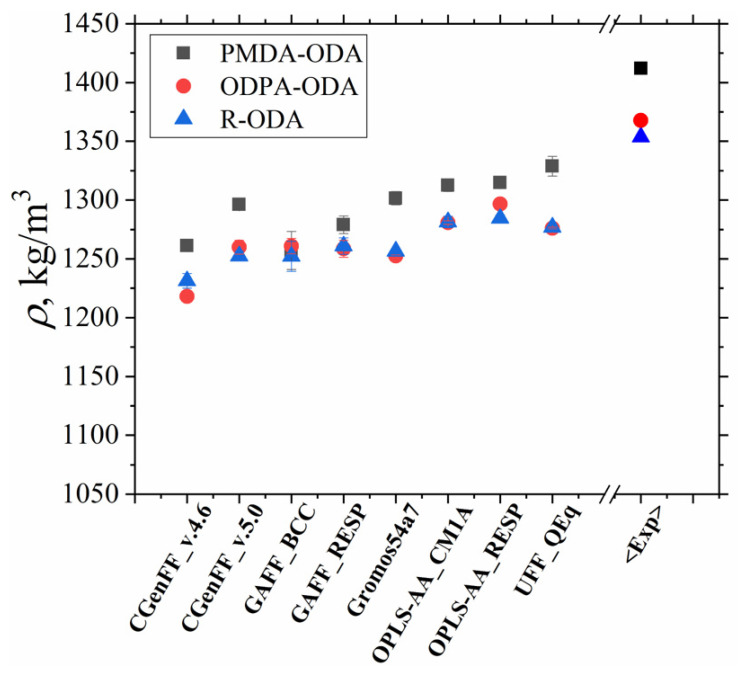
Polymer densities of the studied polyimides at *T* = 290 K.

To further verify the models used in this study, the densities of the three polymers under consideration were calculated at 290 K and compared with the densities obtained experimentally for polyimides synthesized in three different solvents, as shown in Figure 6 and Figure S19 in the Supplementary Materials. The polymer density in the glassy state is primarily determined by a combination of chain characteristics (backbone rigidity and rotational freedom) and intermolecular interactions, which define the efficiency of polymer chain packing at low temperatures. Polymers with rigid backbones are known to have a lower packing density. The experimental density values (Figure 6) show an inverse order compared to the ratio of the polyimide flexibilities (see the values of the persistence lengths in Section 3.1.2). The least flexible polyimide, PMDA-ODA, exhibits the highest density, whereas the more flexible ODPA-ODA and R-ODA demonstrate lower densities. It can be assumed that the barriers to internal rotation and the opportunity to realize strong intermolecular interactions for the polyimide chain packing in a glassy state are decisive.

The data presented indicate that the density values obtained through computer simulations were lower than the experimental results. This discrepancy can be attributed to a significant difference in cooling rates between the simulated and experimental conditions. Specifically, the cooling rate in the simulation was several orders of magnitude higher than that in the experiment, resulting in insufficient time for polymer chain relaxation. Therefore, the simulated values (see the discussion for *T_g_* in Section 3.1.1) must differ from the experimental values but maintain a trend in the same direction as the experimental data. Almost all models (except GAFF_BCC and CGenFF_v.4.6) provide the highest density value for PMDA-ODA. The experimental density ratios, *ρ*(PMDA-ODA) > *ρ*(ODPA-ODA) > *ρ*(R-ODA), were more accurately reproduced by the CGenFF_v.5.0 model. This is not surprising because of the weakening of the intermolecular interactions which allow better chains packing. In contrast to very close *T_g_* values obtained for CGenFF_v.5.0, the densities of the three polyimides are separated and well correlated with the experimental differences between them.

The densities obtained with CGenFF_v.4.6 for three polyimides are lower in comparison with CGenFF-5.0 due to stronger intermolecular (electrostatic) interactions (see discussion in Section Comparison of the Calculated and the Experimental *T_g_*). For ODPA-ODA the density turned out to be sufficiently lower than for the more flexible R-ODA and, as it was shown in the paragraph discussing *T_g_* values, this is due to the realization of the strong intermolecular (stacking type) interactions for ODPA-ODA preventing denser, uniform packing. Conversely, the GAFF_BCC and GAFF_RESP models yielded nearly identical densities for the three polymers studied. Below in Section Free Volume, for these models, the presence of a series of cavities of comparable sizes for the three polyimides in the free volume distributions is discussed with the explanation of the reasons for their formation. The presence of a series of cavities in the free volume distributions obtained for CGenFF_v.4.6, GAFF_BCC and GAFF_RESP models explains also the low-density values for these models.

In conclusion, the best models for describing the relationship between the densities of the polyimides were OPLS-AA_RESP and CGenFF_v.5.0.

##### Coefficient of Volumetric Thermal Expansion, CTE

Our previous study [19] showed that the value of the CTE is practically independent of the thermal history of the sample, that is, the cooling rate at which the sample was cooled to a glassy state, making this characteristic more convenient for comparison with experimental values. CTE and polymer density tend to exhibit an inverse relationship. Polymers with higher densities often have lower CTE values, whereas lower-density polymers tend to have higher CTE values.

The value of CTE was calculated using the temperature dependence of density as $CTE=\frac{1}{V_{0}}{\left(\frac{dV}{dT}\right)}_{p}$, where *V*_0_ is the volume of the system at 290 K and $\frac{dV}{dT}$ is the temperature gradient. The temperature dependence of the CTE values obtained for the PIs is shown in Figure S20 in the Supplementary Materials.

**Figure 7:**
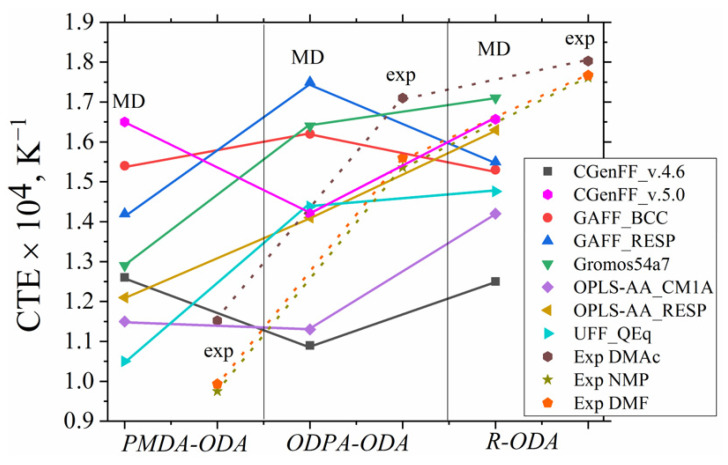
Comparison of the CTE values obtained from the computer simulations and those measured experimentally. Black vertical lines have been added to differentiate the CTE values of the three polyimides. The dashed lines function as visual guides for the experimental CTE values.

The data presented in Figure S20 in the Supplementary Materials show that at low temperatures (300–400 K), the CTE does not change significantly. The average values of CTE for different models and experimental values of CTE in the glassy state (in the temperature range of 300–400 K) are also presented in Table S5 in the Supplementary Materials. For ease of perception, these data are graphically represented in Figure 7.

A comparison of the data obtained in the computer simulations and the experiment shows that all models reproduce (within the interval 1 × 10^4^ K^−1^) the CTE values observed in the experiment. Simultaneously, a more detailed analysis leads to the following conclusions: the Gromos54a7, OPLS-AA_RESP, OPLS-AA_CM1A, CGenFF_v.4.6, and UFF_QEq models reproduce the CTE values for PMDA-ODA better than the others. These models (except for CGenFF_v.4.6) yield the highest and very similar values of PMDA density (Figure 6). If we consider the CTE data for ODPA-ODA, the results of Gromos54a7, GAFF_RESP, GAFF_BCC, UFF_QEq and CGenFF_v.5.0 models can be highlighted by comparing them with the experimental results. The identified models give very close values for these polyimide densities. The CTE values for R-ODA in the Gromos54a7, CGenFF_v.5.0 and OPLS-AA_RESP models are found to converge more closely with the experiment, and as for other polyimides, the listed models provide values of the R-ODA density that are close to the experimental values. It is also worth noting that the qualitative relationship for the CTE values in the polyimide series, both in the simulations and in the experiment, can be determined as follows: CTE(PMDA-ODA) < CTE(ODPA-ODA) < CTE(R-ODA). When the simulation results were compared, a similar CTE trend is observed for the Gromos54a7, OPLS-AA_CM1A and UFF_QEq models. Thus, the model that most accurately describes both characteristics, namely, the CTE values and their relation for the three polymers, is Gromos54a7. When obtaining only the accurate CTE value for each polyimide is required, its own group of models (noted above) can be used. For PMDA-ODA, there are Gromos54a7, OPLS-AA_RESP, OPLS-AA_CM1A, CGenFF_v.4.6, and UFF_QEq models; for ODPA-ODA, there are Gromos54a7, GAFF_RESP, GAFF_BCC, UFF_QEq and CGenFF_v.5.0 models; and for R-ODA, there are Gromos54a7, CGenFF_v.5.0 and OPLS-AA_RESP models.

##### Free Volume

The free volume is the empty space between polymer chains that allows molecular motion. A higher density generally indicates a lower free volume, pointing that the polymer chains are more tightly packed and vice versa. The free volume is crucial for understanding properties such as diffusion, viscosity, and glass transition temperature of a polymer [72,73]. 

**Figure 8:**
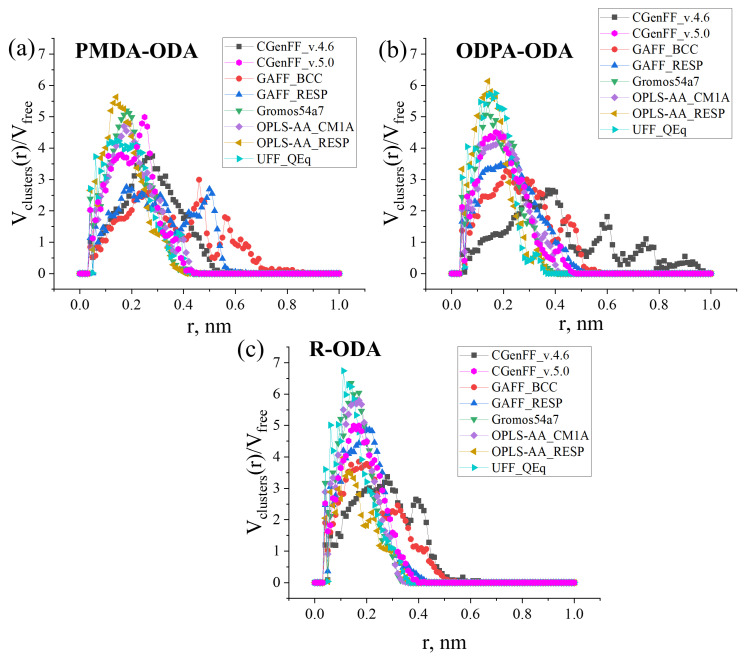
Free volume distribution (% of the total fraction of free volume) of the considered polyimides (**a**) PMDA-ODA, (**b**) ODPA-ODA, and (**c**) R-ODA for different models.

Intra- and intermolecular interactions, which differ in each of the models considered, are expected to have different effects on the free volume distribution after cooling in the glassy state. The free volume distributions were calculated at 290 K and are presented in Figure 8.

The data presented in Figure 8 indicate that, at 290 K, the polyimide samples for the various models considered exhibit comparable sizes of free volume cavities with the highest share in the polymer volume (the first maxima in Figure 8). The most pronounced deviations from the overall set of distributions are observed for the polyimide models parameterized with the CGenFF_v.4.6, GAFF_BCC, and GAFF_RESP models. For these models, the free volume distributions display a primary maximum at relatively large cavity sizes and exhibit multimodal behavior, that is, the presence of several distinct maxima corresponding to cavities of larger sizes. The multimodal distributions of cavity sizes are generally related to different relaxation mechanisms in the glassy state. The distributions of this type were obtained for the CGenFF_v.4.6, GAFF_BCC, and GAFF_RESP models. For CGenFF_v.4.6 it is most pronounced for ODPA-ODA and is explained by the stacking of the phthalimide fragments due to enhanced electrostatic interactions leading to the increase in the activation energies of the mobility of the chain fragments (Figure S16 in the Supplementary Materials). The indicated difference in the activation energy leads to the discrepancy in relaxation times of the chain fragments that is depicted in non-uniformity of polyimide structures in these models, as shown in Figure 8. The multimodal distributions obtained for the GAFF_BCC, and GAFF_RESP models can be explained by the stronger intermolecular (van der Waals) interactions due to the higher values of the parameter ε in these models in comparison with other models, as shown in Figures S21 and S22 in the Supplementary Materials. For this reason, the multimodality is more pronounced for PMDA-ODA as the polyimide having a large a pyrromellitimide cycle in the chain (Figures S23 and S24 in the Supplementary Materials).

The presence of large cavities in the free volume distributions in the indicated models can explain the lower average density at the temperature under consideration, Figure 6. As the backbone flexibility increases in the PMDA-ODA, ODPA-ODA, and R-ODA series, the maxima in the free volume distributions shift to smaller sizes, and the distributions themselves become narrower.

Consequently, the difference in chain flexibility of these three polyimides not only results in differences in their glass transition temperatures but also leads to a reduction in the pore size of the free volume in the glassy state.

### 3.2. Mechanical Properties

**Figure 9:**
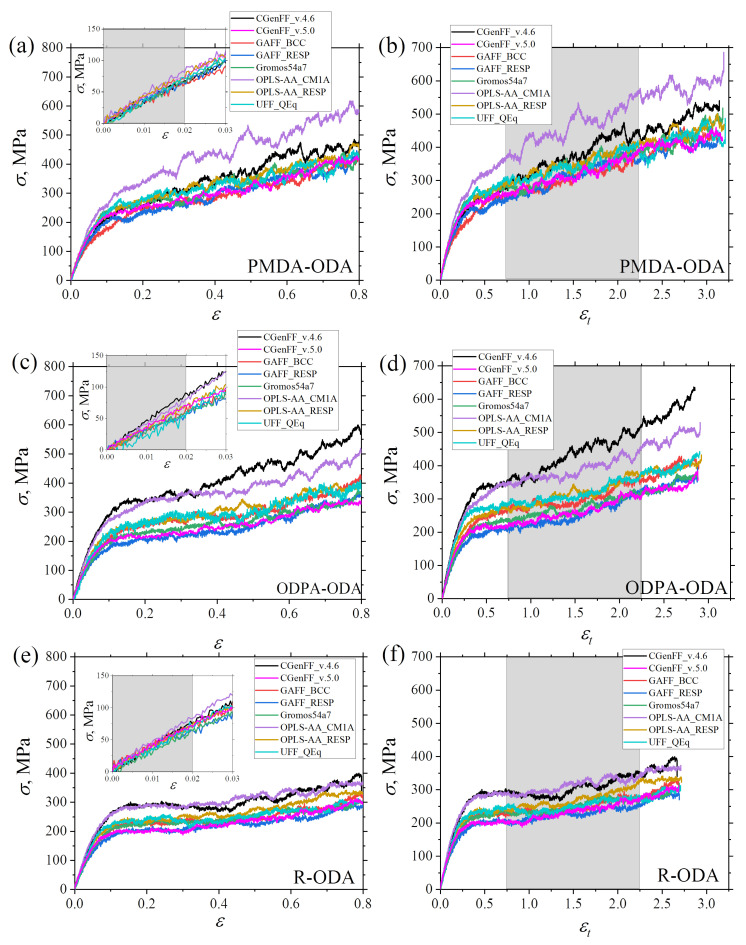
Stress–strain dependences *σ*(*ε*) for PIs (**a**) PMDA-ODA, (**c**) ODPA-ODA and (**e**) R-ODA. Additional insets corresponding to the same dependence but at ε < 2% have also been added to the figures, with the area over which the approximation was performed to determine Young’s modulus highlighted in gray. Dependences *σ*(*ε_t_*) for PIs (**b**) PMDA-ODA, (**d**) ODPA-ODA, and (**f**) R-ODA. The area over which the approximation was performed to determine the yield strength is highlighted in gray.

Three independent configurations obtained after the cooling of the equilibrated systems to 290 K were used to investigate the mechanical properties. The chosen temperature ensured the glassy state of the system under consideration. To calculate the Young’s modulus, yield strength, and strain-hardening modulus [74,75], uniaxial deformation along the X, Y, or Z axes was performed with a strain rate *γ_d_* = 1.8 × 10^8^ s^−1^. In the tensile simulation, the anisotropic $NP_{⊥}T$ ensemble was employed. During the simulation, the periodic box was compressed in the direction perpendicular to the deformation and the pressure $P_{⊥}$ perpendicular to deformation direction was controlled. Conversely, in the direction of deformation, the size of the periodic box was increased, and the system’s compressibility in this direction was maintained at zero. The difference in the strain rates in the experiment and simulations leads to the significant differences in the measured mechanical characteristics in the experiment and simulations. Thus, a qualitative comparison of the experimental and simulation values was performed. During the deformation procedure, the stress–strain relationship *σ*(*ε*) was calculated and analyzed. The description of the uniaxial deformation procedure, the equations to calculate *σ* and *ε* and the description of the calculation of mechanical characteristics are presented in the Supplementary Materials in the Paragraph P5: Mechanical Properties. The stress–strain dependencies obtained are shown in Figure 9.

From the obtained dependences, the values of the Young’s modulus and yield strength were calculated (Table S6 in the Supplementary Materials).

**Figure 10:**
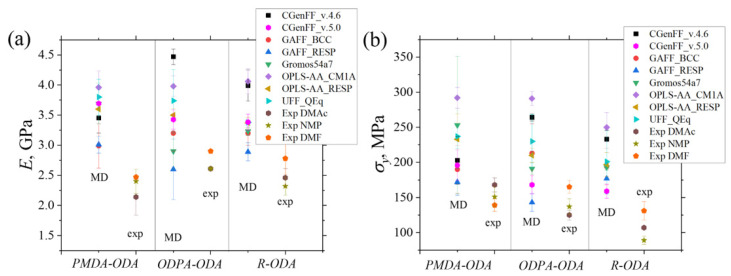
Comparison of values of (**a**) Young’s modulus *E* and (**b**) yield strength *σ_y_* calculated in the simulation and experiment.

The mechanical characteristics were compared with the experimental data, as shown in Figure 10.

The data presented in Figure 10 indicate that the mechanical properties computed through the simulations are predominantly overestimated compared to the experimental results. Table S7 in the Supplementary Materials presents the values of the elastic modulus in the strain-hardening region *G_h_* determined from the dependence of stress on the true relative strain, as shown in Figure 9. It is known from the literature [76,77] that for polymers with less flexible chains, the Young’s modulus and yield strength should be higher than those of more flexible polymers. For the experimentally obtained Young’s modulus in the sequence PMDA-ODPA, ODPA-ODA, and R-ODA, a decrease in the values was observed in the following order: ODPA-ODA > PMDA-ODA > R-ODA. The experimentally obtained ratio between the Young’s moduli was not correlated with the ratio of the chain stiffness values. The Young’s modulus of PMDA-ODA is lower than that of the more flexible polyimide ODPA-ODA. PMDA-ODA polyimide can exhibit a lower Young’s modulus compared to flexible polyimides. This is probably related to the problems of producing thin films with high mechanical characteristics from PMDA-ODA as a stiff-chain polymer. The modulus of PMDA-ODA can be affected by film thickness. Thin films (under 50 μm) can exhibit different relaxation behaviors compared to thicker films, affecting the measured Young’s modulus [78]. In the simulation, the ratio between the Young’s moduli correlating with the ratio between the persistence lengths (Figure 4) is observed for the OPLS-AA_RESP and CGenFF_v.5.0 models.

The ratio between the experimental values of the yield strength depending on the solvent either correlates with the ratio for the polyimide stiffness or is violated in the same way as for the modulus. Since the yield strength strongly depends on the intermolecular interactions, it leads to differences in structure formation in different solvents and this may explain the observed behavior. The ratio of yield strength that correlates with the ratio of the chain stiffness is obtained for the OPLS-AA_RESP and CGenFF_v.5.0 models as in the case of Young’s moduli.

The elastic strain yield, *ε_y_*, was calculated using both computer simulations and experiments as σ_y_/E [79]. The values are shown in Figure S25a in the Supplementary Materials. This mechanical property is related to the engineering strain accumulated during elastic deformation prior to irreversible plastic flow. The results indicated that the calculated simulation values of *ε_y_* are in the same range as the experimental *ε_y_* values. The typical simulation values were between 0.06 and 0.09. The OPLS-AA_CM1A, OPLS-AA_RESP and CGenFF_v.5.0 models can reproduce the ratio shown in the experiment for *ε_y_*(PMDA-ODA) > *ε_y_*(ODPA-ODA)> *ε_y_*(R-ODA).

It should be noted that in our all-atom simulation, it is important to note that bond breaking does not occur during plastic deformation. This limitation may affect the comparability of polymer matrix deformation between computer simulations and experimental data. A reactive molecular dynamics force field could be more suitable for accurately representing polymer behavior during deformation [80]. Furthermore, all-atom molecular dynamics simulations are limited in comparison to united-atom or coarse-grained models when it comes to increasing the molecular weight of the polymer system. This limitation is significant for considering the entanglement effect, which influences the plastic deformation of polymer systems [81,82].

A detailed analysis of the strain-hardening modulus (Table S7 in the Supplementary Materials) shows that the values of *G_h_* follow the order *G_h_*(PMDA-ODA) > *G_h_*(ODPA-ODA) > *G_h_*(R-ODA), which is similar to the order of the Kuhn segment values *b* of PI, *b*(PMDA-ODA) > *b*(ODPA-ODA) > *b*(R-ODA). This result is supported by the recent theory of Hoy et al. [83], where it was shown that the value of *G_h_* is proportional to *b* and is equal to $S_{c}\left(u_{0}/l_{0}^{3}\right)B^{3}$, where statistical segment length $B=\sqrt{l_{0}b}$ (where *l*_0_ is the backbone bond length and *b* is Kuhn segment), the intermonomer binding energy is *u*_0_, and the incremental elastic strain required to activate Kuhn-segment-scale plastic rearrangements is *S_c_*. The calculated value of the strain-hardening modulus *G_h_* was incorporated into different simulation models to estimate the mechanical stiffness of the studied polymers. From the simulation, the material stiffness might be estimated by the calculation of the so-called Considere’s criterion *G_h_*/*σ_y_*, which is useful for a characterization of the material transition from yield to hardening during deformation [84], that is often related to the neck formation as a result of the deformation when *G_h_*/*σ_y_* < 1/3. As shown in Figure S25b in the Supplementary Materials, the results indicate that *G_h_*/*σ_y_* is greater for PMDA-ODA in most simulation models (except for OPLS-AA_RESP and UFF_QEq). This supports the notion that less flexible PMDA-ODA has transitioned from the yield to the hardening regime with a narrow plastic plateau, as shown in Figure 9. Such behavior is likely to be associated with the substantially higher material strength of PMDA-ODA compared to that of ODPA-ODA and R-ODA polyimides.

## 4. Conclusions

For the first time, in the current study, microsecond-scale all-atom computer simulations were performed to perform systematic investigation of the influence of the force field type selected in computer simulations on the thermal and mechanical properties of three polyimides with different chemical structures of the dianhydride fragments PMDA-ODA, ODPA-ODA, and R-ODA. Eight models of thermoplastic polyimides were considered based on the five force fields most commonly used in polymer simulations: GAFF, OPLS-AA, CGenFF, Gromos54a7, and UFF. Two different types of partial charges were considered for the two force fields GAFF (AM1-BCC and HF/6-31G*(RESP)) and OPLS-AA (CM1A and HF/6-31G*(RESP)). Because the cooling and deformation rates in the experiment and simulation differ significantly, most simulated characteristics can be compared only qualitatively with the experiment.

The influence of the thermodynamics and kinetic flexibility of the polyimide chains on the thermal and mechanical properties was evaluated by applying a detailed comparison of the simulation results with the experimental data. The correlation between the ratio of the glass transition temperature values and mechanical properties (Young’s modulus, yield strength, and strain-hardening modulus) with the ratio of the thermodynamic flexibility (persistence length and Kuhn segment) for the considered polyimide models was established for a part of the models considered. The thermodynamic flexibilities are correctly described in all models owing to the close values of the bonded parameters in the corresponding force fields. Polymer models for which the absence of the correlation between thermodynamic flexibility and thermomechanical properties was obtained can be explained by an incorrect estimation in these force fields of the kinetic flexibilities determined by a combination of intramolecular barriers and intermolecular interactions.

Based on the analysis of the ratios of glass transition temperatures for the three investigated polyimides, the experimentally observed sequence *T_g_*(PMDA-ODA) > *T_g_*(ODPA-ODA) > *T_g_*(R-ODA) was reproduced by the simulations for all considered models, with the exception of those employing the CGenFF_v.4.6 model.

The density values from the computer simulations were lower than those of the experimental results. The experimental density ratios, *ρ*(PMDA-ODA) > *ρ*(ODPA-ODA) > *ρ*(R-ODA), were best reproduced by the OPLS-AA_RESP and CGenFF_v.5.0 models.

Our analysis of the simulation data for mechanical properties revealed trends in model performance. The mechanical properties decreased consistently across the simulations in the order of PMDA-ODA > ODPA-ODA > R-ODA. In the simulation, the ratio between Young’s modulus and yield strength correlating with the ratio between thermodynamic flexibilities was observed for the OPLS-AA_RESP and CGenFF_v.5.0 models. A straight correlation was found for the ratio of the strain-hardening modulus values and the ratio of the thermodynamic flexibilities in the current simulation. It was found that all the considered models were capable of reproducing the relationship of the Kuhn segments calculated from the simulations.

Because the CTE values are practically independent of the thermal history of the sample [19], they can be compared with experimental results, not only qualitatively but also quantitatively. The experimental trend of increasing CTE in the series PMDA-ODA < ODPA-ODA < R-ODA is also mirrored in the simulations using Gromos54a7, OPLS-AA_RESP, and UFF_QEq models. Gromos54a7 is the most accurate model for describing not only the ratio between the CTE of these polymers but also their values for each polyimide. The accurate CTE value for each polyimide can be reproduced for PMDA-ODA using OPLS-AA_RESP, OPLS-AA_CM1A, CGenFF_v.4.6, and UFF_QEq models. For ODPA-ODA, the GAFF_RESP, GAFF_BCC, and OPLS-AA_RESP models can be used. For R-ODA, the GAFF_RESP, GAFF_BCC and CGenFF_v.5.0 models can be suggested.

The proposed approach for simulating the thermal and mechanical properties of thermoplastic polyimides with varying dianhydride fragment structures could be applied to simulate other polyimides to qualitatively and even quantitatively (CTE) predict experimental data using all-atom molecular dynamics simulations. A detailed understanding of the characteristics of intra- and intermolecular interactions in polyimides will enable the identification of optimal strategies for tailoring their chemical structures and the development of approaches to control their thermal and mechanical properties.

## Data Availability

The data presented in this study are available upon request from the corresponding author. The data are not publicly available due to the large size of simulation trajectories.

## Supplementary Materials

The following supporting information can be downloaded at: https://www.mdpi.com/article/10.3390/polym18131624/s1. See the Supplementary Materials for more information on difference in functional form of potential types in considered force fields, the criteria for system equilibrium, average chain size of polymers, comparison of radial distribution functions, comparison of values of partial charges and Lennard–Jones parameters of considered force fields, comparison of methods for determining the glass transition temperature, details of experimental density, calculated parameters for local chain mobility, methods for calculating persistence length, and calculation of mechanical properties. Figure S1. Time dependence of average end-to-end distance, radius of gyration, ratio between at *T* = 1200 K. Figure S2. Mean squared displacement of the center of mass polymer chain of PMDA-ODA, ODPA-ODA, and R-ODA polyimides in different considered models at *T* = 1200 K. Figure S3. Average values of end-to-end distance, radius of gyration, characteristic ratio, and contour length of the different models for the three PIs at *T* = 1200 K. Figure S4. Comparison of the glass transition temperatures obtained from the simulation using different methods: standard, Hyperbola method, Shapiro method, MSD method, and the experiment of studied polyimide PMDA-ODA, ODPA-ODA, and R-ODA. Figure S5. Temperature dependences of the logarithm of the MSD, averaged over three samples obtained for different models of studied polyimide PMDA-ODA, ODPA-ODA, and R-ODA. Figure S6. The experimental values of *T_g_*. Figures S7–S9. Radial distribution functions of centroid of carbon atoms between the diamine-diamine, dianhydride-dianhydride, diamine-dianhydride and all_aromatic-all_aromatic of fragment groups of polyimide PMDA-ODA (S7), ODPA-ODA (S8), and R-ODA (S9) for different all-atom models at T = 1200 K. Figure S10. Radial distribution functions of centroid of carbon atoms between the diamine-diamine, dianhydride-dianhydride, diamine-dianhydride and all_aromatic-all_aromatic of fragment groups of polyimide ODPA-ODA for CGenFF_v.4.6, CGenFF_v.5.0 and CGenFF_v.4.6 with partial charges values models at T = 1200 K scaled in the 0.8-time. Figure S11. Partial charge values of the middle repeating unit (from 2 to 11) of the studied polyimides PMDA-ODA, ODPA-ODA, and R-ODA. Figure S12. The chemical structure of the repeating unit of PMDA-ODA, ODPA-ODA and R-ODA. Figure S13. Virtual bond vector autocorrelation functions (BACF) of polyimide PMDA-ODA, ODPA-ODA, and R-ODA. Figure S14. The ratio between the Kuhn segment and the persistence length of the studied PIs for different models. Figure S15. Temperature dependence of the average relaxation times for the orientational mobility of the normal vectors PH_1,2_ for different models of for the PI samples: PMDA-ODA, ODPA-ODA, and R-ODA. Figure S16. Activation energy values of the normals to the surface of various heterocyclic rings of thermoplastic polyimides located in the dianhydride and the diamine fragment of the repeating unit of the thermoplastic polyimides PMDA-ODA. ODPA-ODA, and R-ODA. Figure S17. Critical temperatures *T_c_* and *T*_0_ determined using the MCT coupled mode theory and the VFT equation, calculated from the temperature dependencies of the local orientation relaxation time of unit normals to the surface of heterocyclic rings in the dianhydride moiety of the PMDA-ODA repeating unit of polyimides–PH_3_, ODPA-ODA–PH_4,5_, and R-ODA–PH_6,8_ and PH_7_. Figure S18. Values of critical temperatures *T_c_* and *T*_0_ determined using the MCT coupled mode theory and the VFT equation, calculated from the temperature dependencies of the local orientation relaxation time of unit normals PH_1,2_ to the surface of heterocyclic rings in the diamine fragment of the repeating unit of polyimides PMDA-ODA, ODPA-ODA, and R-ODA. Figure S19. Experimental values of density. Figure S20. Temperature dependence of the CTE of PIs PMDA-ODA, ODPA-ODA, and R-ODA in different force fields. Figures S21 and S22. Per-atom Lennard–Jones ε of the dianhydride (S21) and diamine (S22) rings atoms of studied polyimide PMDA-ODA, ODPA-ODA, R-ODA for different all-atom models. Figures S23 and S24. Radial distribution functions of centroid of carbon atoms between the diamine-diamine, dianhydride-dianhydride, diamine-dianhydride and all_aromatic-all_aromatic of fragment groups of considered polyimides PMDA-ODA, ODPA-ODA and R-ODA for GAFF_BCC (S23) and GAFF_RESP (S24) models at T = 1200 K. Figure S25. The mechanical characteristics of studied polyimide calculated from the values of Young’s modulus, yield strength and strain-hardening modulus: elastic yield strain and Considere’s criterion. Table S1. Functional form of potential types of interactions in the considered force fields. Table S2. Computational performance of atomistic models of polyimide samples at T = 1200 K in molecular dynamics simulations. Table S3. Glass transition temperatures of the studied PIs for the considered models. Table S4. Persistence length of the studied polyimides obtained via computer simulation. Table S5. Average values of CTE for different force fields and experimental values of CTE in the glassy state. Table S6. Mechanical characteristics (Young’s modulus and yield strength) of the studied polyimides obtained via computer simulations. Table S7. Mechanical characteristics (strain-hardening moduli) of the studied polyimides obtained by computer simulations.
