# Supplementary material for: Investigation of the Influence of Thermodynamic and Kinetic Flexibility of Polymer Chains in Thermoplastic Polyimides on Their Thermal and Mechanical Properties: Experiment and All-Atom Computer Simulations
Source: Polymers (Basel). 2026 Jun 30;18(13):1624. doi: 10.3390/polym18131624 (PMC13363942; doi:10.3390/polym18131624)
Supplement: Supplementary file 1 [file polymers-18-01624-s001.zip › polymers-4336611-supplementary.pdf]

Supplementary Materials

Investigation of the Influence of Thermodynamic and Kinetic Flexibility of Polymer Chains in Thermoplastic Polyimides on Their Thermal and Mechanical Properties: Experiment and All-Atom Computer Simulations

Victor M. Nazarychev [1,*], Natalia V. Lukasheva [1], Andrei L. Didenko [1], Vera E. Sitnikova [2], Ivan V. Abalov [1] and Vladislav V. Kudryvtsev [1]

[1] Branch of Petersburg Nuclear Physics Institute Named by B.P. Konstantinov of the National Research Centre «Kurchatov Institute»—Institute of Macromolecular Compounds, St. Petersburg 199004, Russia

[2] The Center for Chemical Engineering, ITMO University, Saint Petersburg 197101, Russia

S1. Potential energy functional forms for considered force fields

In general, the total potential energy E_{pot} of a classical force field is given as:

$$E_{pot} = E_{bond} + E_{angle} + E_{dih} + E_{imp} + E_{vdw} + E_{coul},$$

where E_{bond}, E_{angle}, E_{dih} and E_{imp} are the energies of bond, angle, dihedral and improper interactions, E_{vdw} is the energy of the van der Waals interactions, and E_{coul} is the energy of the electrostatic interactions. Each atomistic force field for polyimides possesses a distinct set of potential functions and corresponding parameters to characterize system interactions. The specific functional form of these force fields can be referenced in the Gromacs manual, for details please see [1]. Table S1 provides a summary of the potential function's functional form for the force field examined.

Table S1. Functional form of potential types of interactions in the considered force fields.

<table>
<tr><th rowspan="2">Potential type</th><th colspan="5">Force fields</th></tr>
<tr><th>CGenFF</th><th>GAFF</th><th>Gromos54a7</th><th>OPLS-AA</th><th>UFF</th></tr>
<tr><td>Lennard-Jones</td><td colspan="2">(sigma/epsilon form): $U_{\mathrm{LJ}}(r) = 4\varepsilon\left[\left(\frac{\sigma}{r}\right)^{12} - \left(\frac{\sigma}{r}\right)^{6}\right]$</td><td>(C6/C12 form, comb-rule 1): $U_{\mathrm{LJ}}(r) = \frac{C_{12}}{r^{12}} - \frac{C_6}{r^6}$</td><td colspan="2">(sigma/epsilon form): $U_{\mathrm{LJ}}(r) = 4\varepsilon\left[\left(\frac{\sigma}{r}\right)^{12} - \left(\frac{\sigma}{r}\right)^{6}\right]$</td></tr>
<tr><td>Electrostatics</td><td colspan="5">$U_{\mathrm{elec}}(r) = \frac{1}{4\pi\varepsilon_0}\frac{q_i q_j}{r}$</td></tr>
<tr><td>Bond</td><td colspan="2">Harmonic bond (funct 1): $U(r) = \frac{1}{2}k_b(r - b_0)^2$</td><td>GROMOS-96 fourth-power bond (funct 2): $U(r) = \frac{1}{4}k_b\left(r^2 - {b_0}^2\right)^2$</td><td colspan="2">Harmonic bond (funct 1): $U(r) = \frac{1}{2}k_b(r - b_0)^2$</td></tr>
<tr><td>Angle</td><td>Urey-Bradley angle (funct 5): $U = \frac{1}{2}k_\theta(\theta - \theta_0)^2 + \frac{1}{2}k_{\mathrm{UB}}\left(r_{13} - r_{13,0}\right)^2$</td><td>Harmonic angle (funct 1): $U(\theta) = \frac{1}{2}k_\theta(\theta - \theta_0)^2$</td><td>GROMOS-96 cosine-harmonic angle (funct 2): $U(\theta) = \frac{1}{2}k_\theta(\cos\theta - \cos\theta_0)^2$</td><td>Harmonic angle (funct 1): $U(\theta) = \frac{1}{2}k_\theta(\theta - \theta_0)^2$</td><td>GROMOS-96 cosine-harmonic angle (funct 2): $U(\theta) = \frac{1}{2}k_\theta(\cos\theta - \cos\theta_0)^2$</td></tr>
<tr><td>Proper dihedral</td><td colspan="2">Periodic proper dihedral (multiple allowed) (funct 9): $U(\varphi) = \sum_k k_{\varphi,k}\left[1 + \cos\left(n_k\varphi - \varphi_{s,k}\right)\right]$</td><td>Periodic proper dihedral (funct 1): $U(\varphi) = k_\varphi[1 + \cos(n\varphi - \varphi_s)]$</td><td>Ryckaert-Bellemans dihedral (funct 3): $U(\psi) = \sum_{n=0}^{5} C_n\cos^n(\psi)$</td><td>Periodic proper dihedral (funct 1): $U(\varphi) = k_\varphi[1 + \cos(n\varphi - \varphi_s)]$</td></tr>
<tr><td>Improper dihedral</td><td>Harmonic improper dihedral (funct 2): $U(\xi) = \frac{1}{2}k_\xi(\xi - \xi_0)^2$</td><td>Periodic improper dihedral (funct 4): $U(\varphi) = k_\varphi[1 + \cos(n\varphi - \varphi_s)]$</td><td>Harmonic improper dihedral (funct 2): $U(\xi) = \frac{1}{2}k_\xi(\xi - \xi_0)^2$</td><td>Periodic improper dihedral (funct 4): $U(\varphi) = k_\varphi[1 + \cos(n\varphi - \varphi_s)]$</td><td>Harmonic improper dihedral (funct 2): $U(\xi) = \frac{1}{2}k_\xi(\xi - \xi_0)^2$</td></tr>
</table>

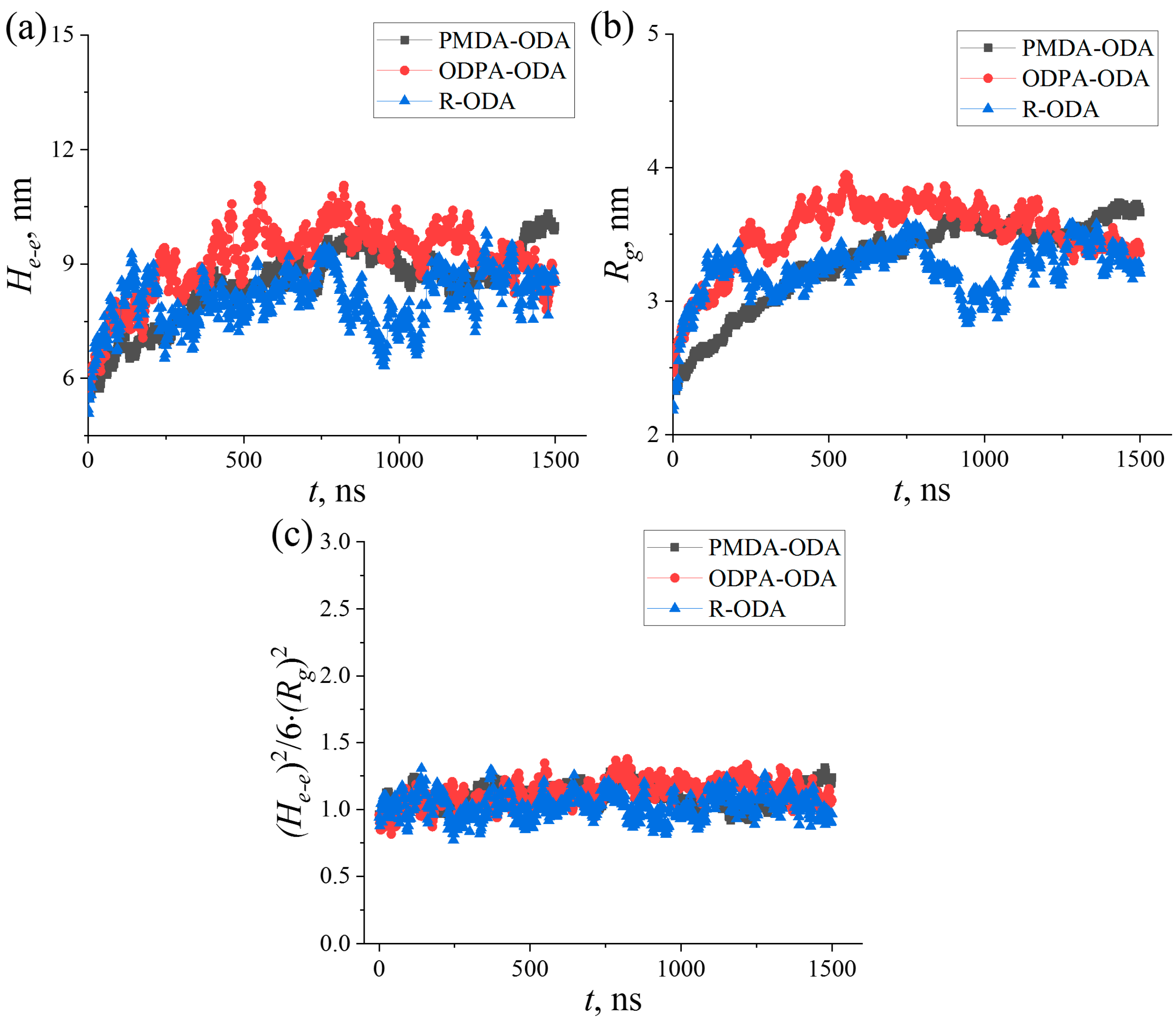

Figure S1. Time dependence of average (a) end-to-end distance (**H_{e-e}**), b) radius of gyration (**R_g**), c) ratio between (**H_{e-e}**/**R_g**)^2/6 at *T*=1200 K.

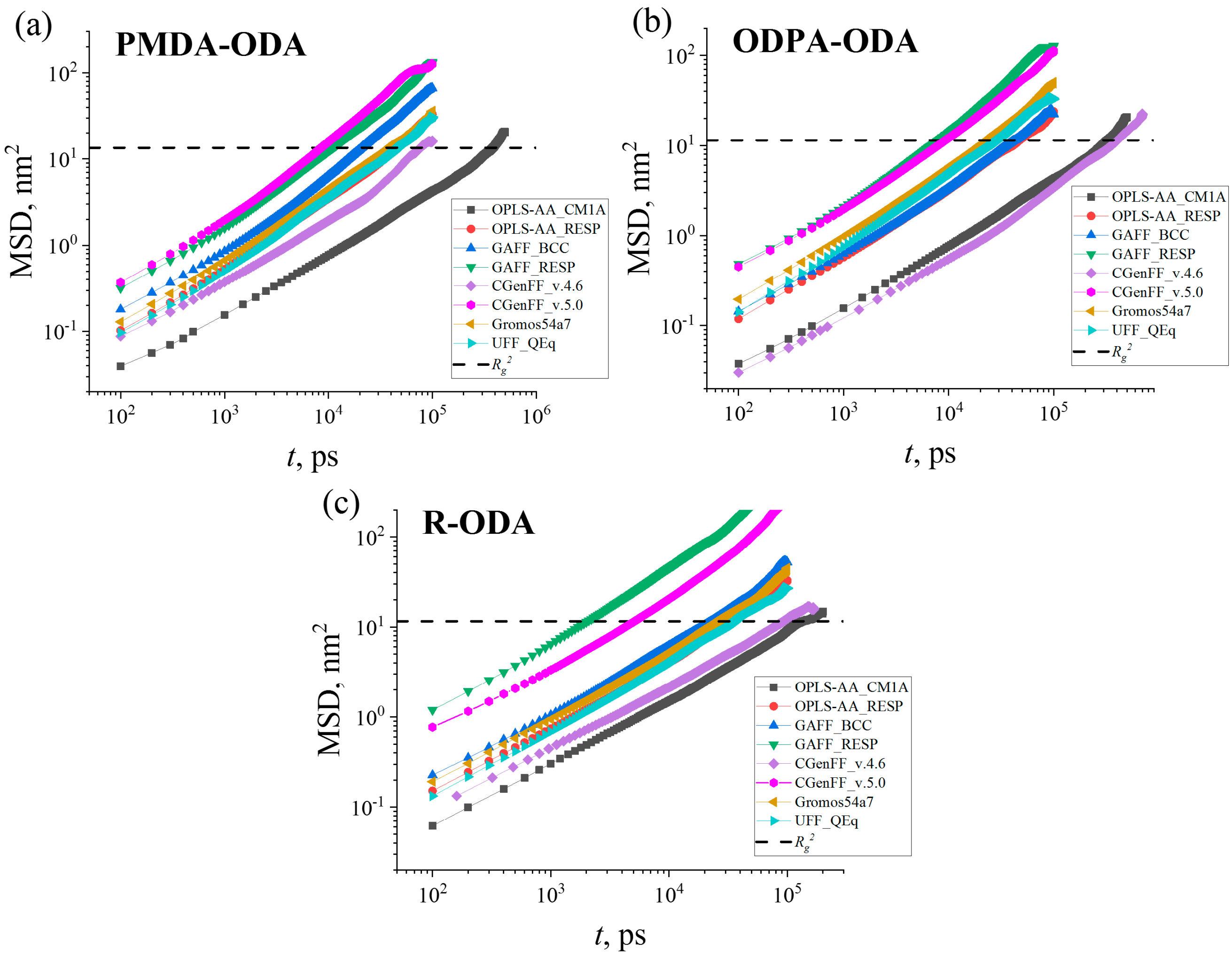

Figure S2. Mean squared displacement of the center of mass polymer chain of (a) PMDA-ODA, (b) ODPA-ODA, and (c) R-ODA in different considered models at T = 1200 K.

Table S2. Computational performance (ns/day) of eight atomistic models of polyimide samples at T = 1200 K in molecular dynamics simulations performed with Gromacs (version 2022). The GROMACS simulations were carried out on an AMD EPYC 9654 processor with 96 physical cores and 192 logical processors, together with two GeForce RTX 4080 SUPER Phoenix GS 16GB GPUs (Gainward, China) using 32 processors on each GPU video card for one calculation run.

Polymer	CGenFF_v.1.0	CGenFF_v.5.0	Gromos54a7	GAFF_BCC	GAFF_RESP	OPLS-AA_CM1A	OPLS-AA_RESP	UFF_QEq
PMDA-ODA	433 ± 9	442 ± 3	448 ± 1	417 ± 1	417 ± 4	416 ± 2	415 ± 7	441 ± 3
ODPA-ODA	383 ± 4	396 ± 1	408 ± 2	370 ± 1	373 ± 3	364 ± 1	373 ± 1	397 ± 4
R-ODA	346 ± 3	352 ± 4	372 ± 4	335 ± 4	340 ± 3	328 ± 1	331 ± 1	358 ± 1

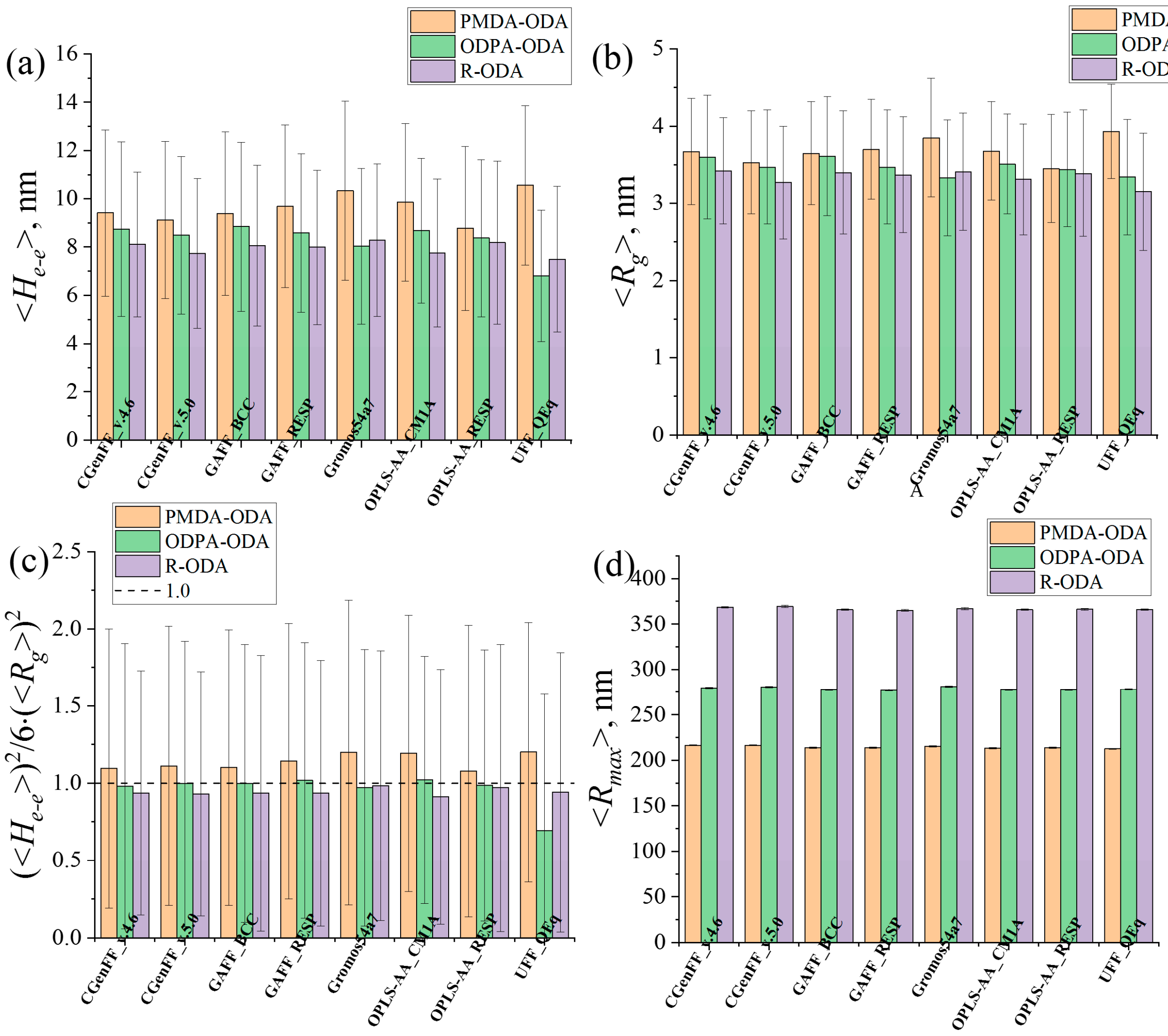

Figure S3. Average values of (a) end-to-end distance $<H_{e\text{-}e}>$, (b) radius of gyration $<R_g>$, (c) characteristic ratio $(<H_{e\text{-}e}>)^2/(6\cdot(<R_g>)^2)$, and (d) contour length $<R_{max}>$ of the different models for the three PIs at $T = 1200$ K. The dotted line in (c) indicates that $(<H_{e\text{-}e}>)^2/(6\cdot(<R_g>)^2)$ is equal to 1.

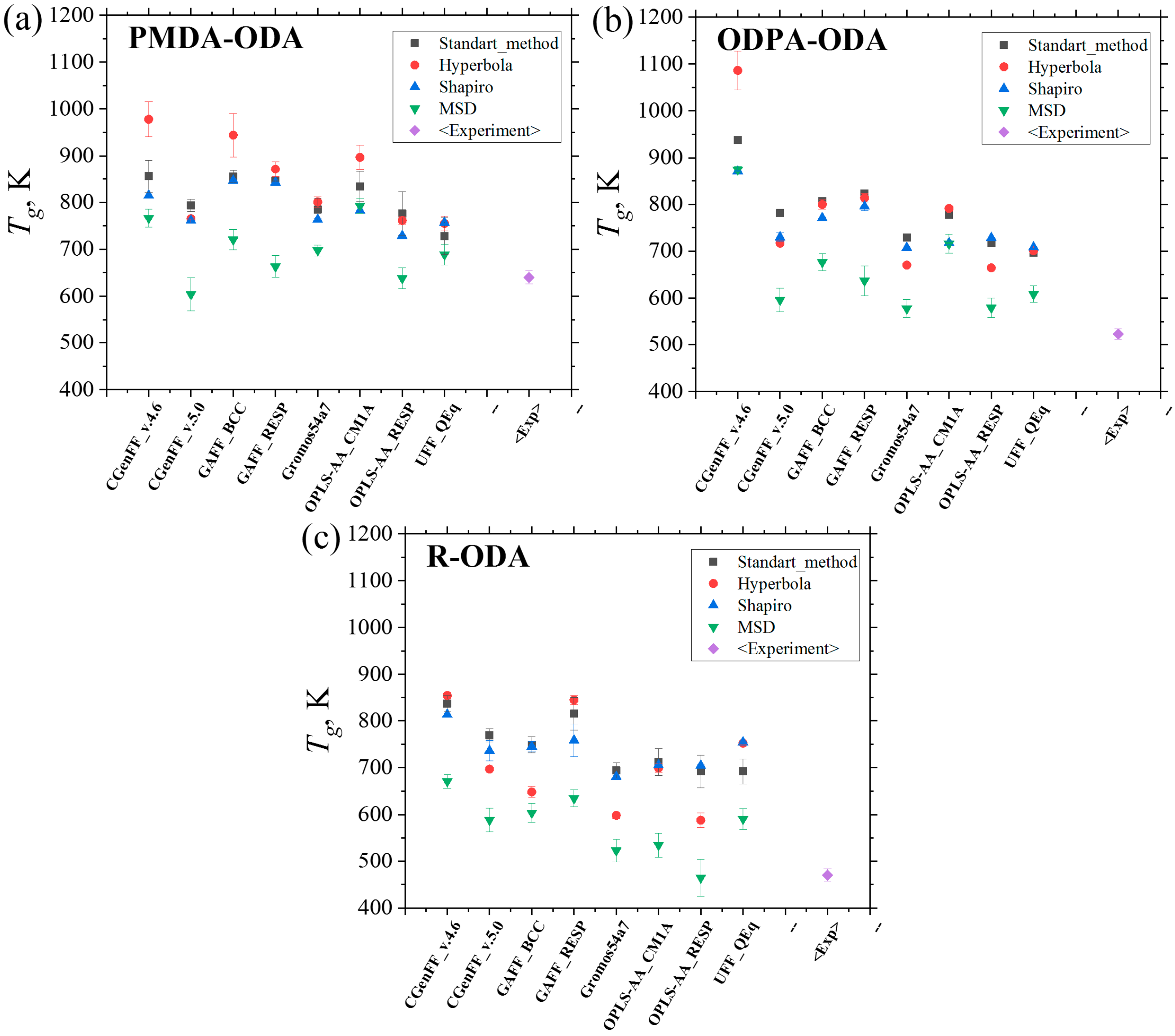

Figure S4. Comparison of the glass transition temperatures obtained from the simulation using different methods: standard – from the main text of the manuscript – black symbols, Hyperbola method [2] (red symbols), Shapiro method [3] (blue symbols), MSD method [4] (green symbols), and the experiment (purple symbols) of studied polyimide (a) PMDA-ODA, (b) ODPA-ODA, and (c) R-ODA. "MSD" T_g values were obtained from the MSD curves shown in Figure S5. The error value is calculated as the root mean square deviation from the mean value, based on three samples.

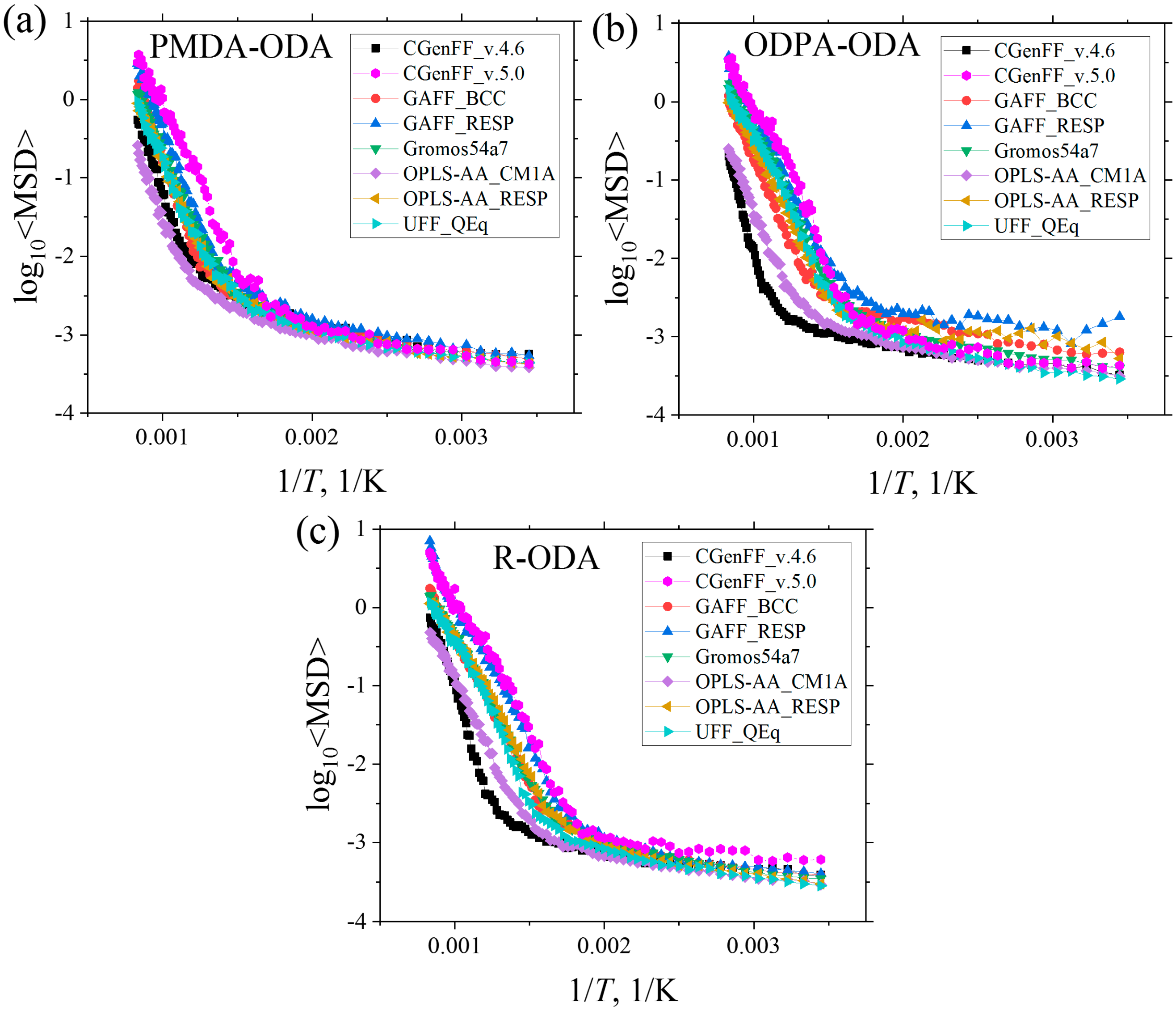

Figure S5. Temperature dependences of the logarithm of the MSD, averaged over three samples obtained for different models of studied polyimide (a) PMDA-ODA, (b) ODPA-ODA, and (c) R-ODA.

Table S3. Glass transition temperatures of the studied PIs for the considered models.

	T_g, K		
Model / Polyimide	PMDA-ODA	ODPA-ODA	R-ODA
CGenFF_v.4.6	856±35	938±2	837±17
CGenFF_v.5.0	794±13	781±6	768±14
GAFF_BCC	855±21	807±36	749±17
GAFF_RESP	846±5	823±18	815±35
Gromos54a7	785±24	729±4	694±17
OPLS-AA_CM1A	834±35	777±15	712±29
OPLS-AA_RESP	776±47	718±26	692±35
UFF_QEq	728±39	697±10	693±27

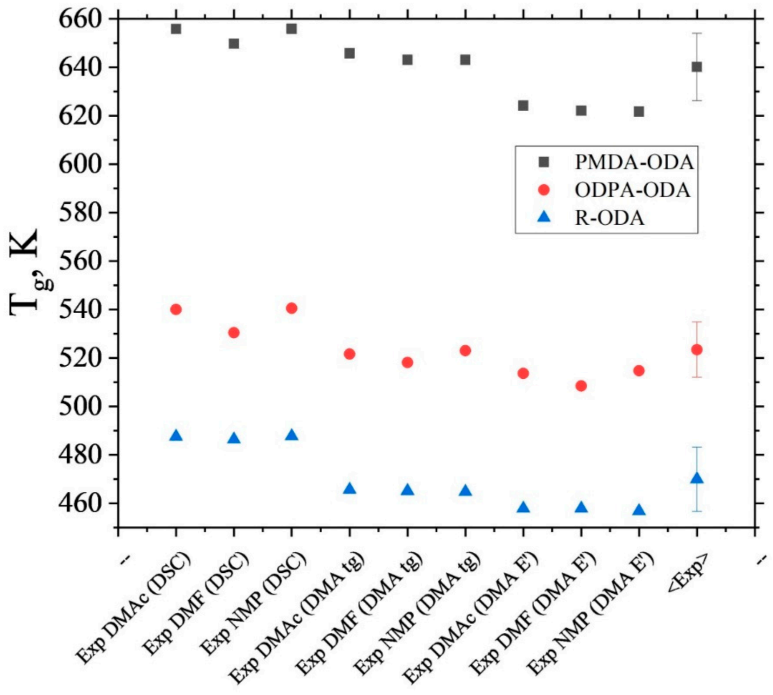

Figure S6. The experimental values of T_g are listed in Table S1. Calculated glass transition temperatures for the three PIs using different models. The standard deviation from the mean value is given as an error. The results were measured in previous study [5].

S2. RDF calculation

The radial distribution function (RDF) g(r) is the average number of *B* sites found in a spherical shell of radius *r* and thickness Δr around an *A* site, normalized by the number expected for a uniform distribution at the same bulk density. It is computed as

$$g_{AB}(r) = \frac{\langle n_{AB}(r)\rangle}{\rho_B \cdot 4\pi r^2 \cdot \Delta r \cdot N_A} \qquad \text{(S1)}$$

where $\langle n_{AB}(r)\rangle$ is the average count of *A*–*B* pairs whose separation falls in the shell [r, $r+\Delta r$], N_A is the number of A sites, and $\rho_B = \langle N_B\rangle/\langle V\rangle$ is the mean number density of *B* sites in the box. The shell volume is evaluated as

$$V_{shell} = \frac{4}{3}\pi({r_{out}}^3 - {r_{in}}^3) \qquad \text{(S2)}$$

A sharp first peak g(r) near 3.5–4.0 Å in the centroid RDF is a signature of cofacial (parallel) π–π stacking, while a broad feature near 5–7 Å corresponds to neighboring rings that are offset or edge-on rather than stacked [6].

Each benzene ring was then classified as a marker of the imide group:

1. dianhydride_imide: a benzene ring fused to an imide ring (sharing two or more atoms with it): the imide-bearing aromatic rings of the dianhydride (PMDA-ODA: PH_3; ODPA-ODA: (PH_4 and PH_5); R-ODA: (PH_6 and PH_8).
2. diamine: a benzene ring not fused to an imide but bonded to an imide nitrogen: the ODA phenylene rings (PH_1 and PH_2), common to all three polymers.
3. dianhydride_linker: a benzene ring bonded only through ether oxygens (no imide) in the central non-imide ring of R-ODA (PH_7).

Only the carbon atoms of the classified benzene rings were included in the RDF; hydrogen and the heteroatoms (N, O) were not included because π–π stacking is an interaction between the aromatic carbon π-systems. The rings were collected into two fragment groups for comparison:

1. diamine - the carbon atoms of the diamine (ODA) phenylene rings.
2. dianhydride - the carbon atoms of the dianhydride rings (dianhydride_imide together with dianhydride_linker).
3. all_aromatic, is the union of the two.

For each ring the centroid is the arithmetic mean of the positions of its six aromatic carbon atoms,

$$R_c = \frac{1}{n}\sum_{k=1}^{n} r_k \qquad \text{(S3)}$$

with n = 6. The RDF is then evaluated between the centroids of the rings in group A and the centroids of the rings in group B. Because each ring is reduced to a single point, the centroid RDF reports the ring-to-ring stacking distance cleanly and its first peak position is the stacking separation, which is the primary π–π metric.

The index of the chain to which each ring belongs was calculated, and any pair of sites on the same chain was removed before binning. This intermolecular restriction isolates the interchain stacking from the fixed intrachain ring geometry.

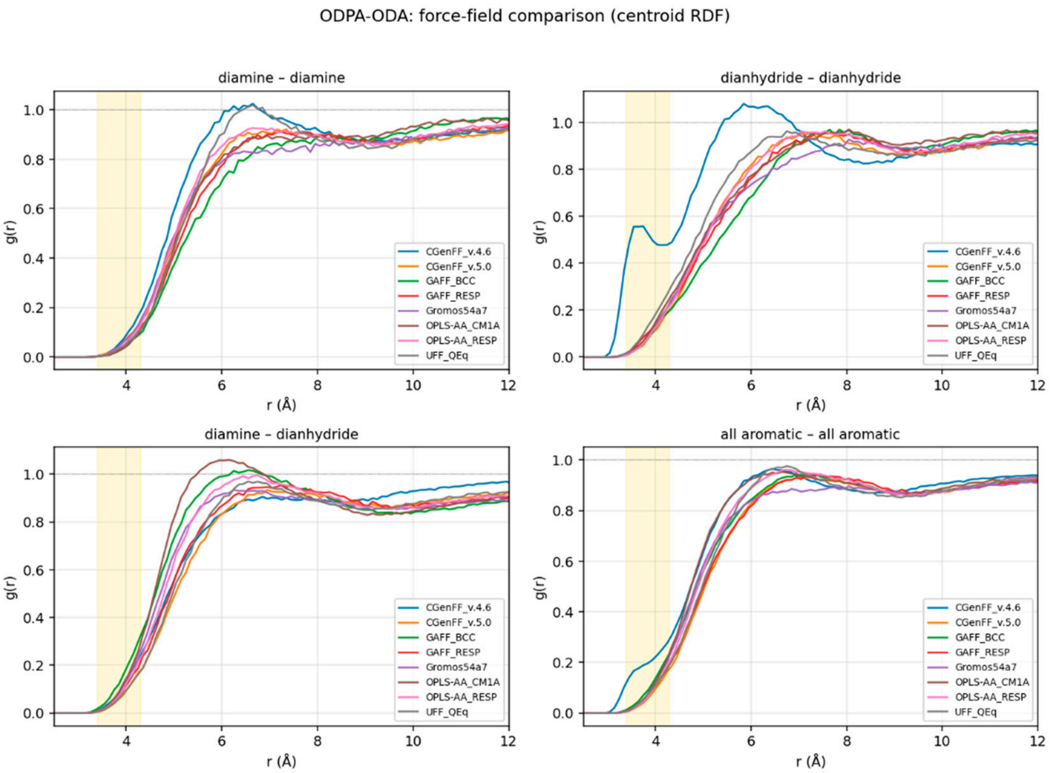

Figure S7. Radial distribution functions (RDFs) of centroid of carbon atoms between the diamine-diamine (top, left), dianhydride-dianhydride (top, right), diamine- dianhydride (bottom, left) and all_aromatic - all_aromatic (bottom, right) of fragment groups of polyimide ODPA-ODA for different all-atom models at T = 1200 K.

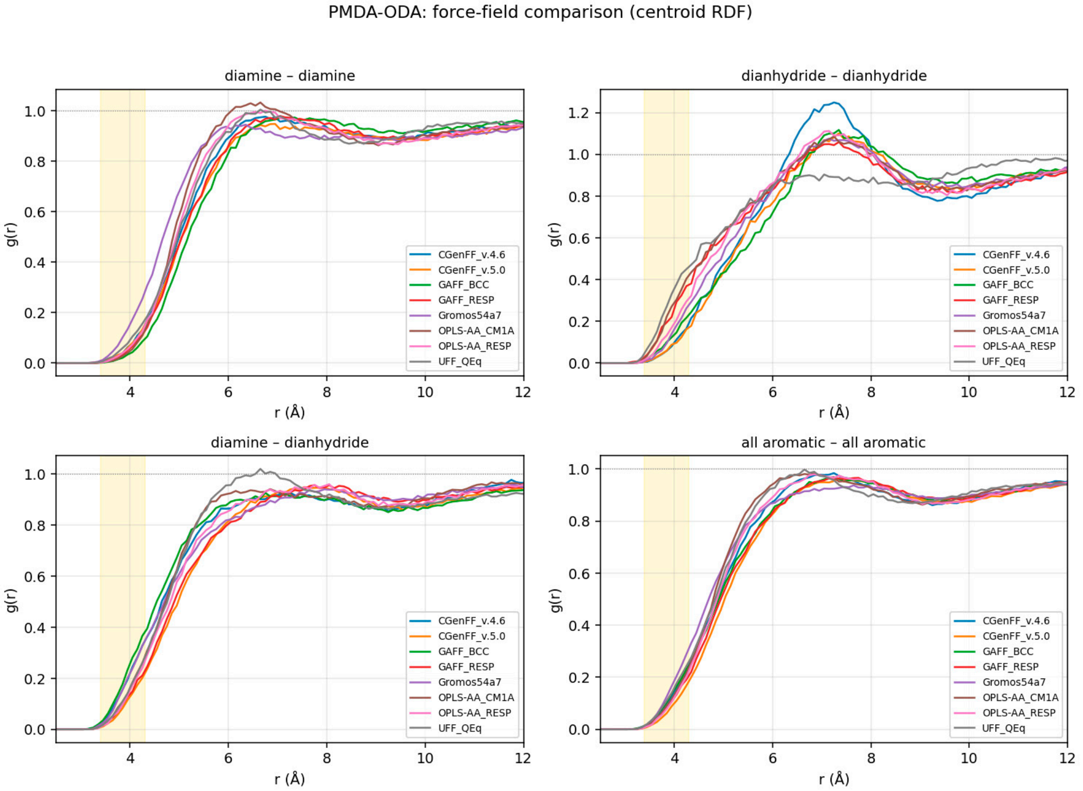

Figure S8. Radial distribution functions (RDFs) of centroid of carbon atoms between the diamine-diamine (top, left), dianhydride-dianhydride (top, right), diamine- dianhydride (bottom, left) and all_aromatic - all_aromatic (bottom, right) of fragment groups of polyimide PMDA-ODA for different all-atom models at T = 1200 K.

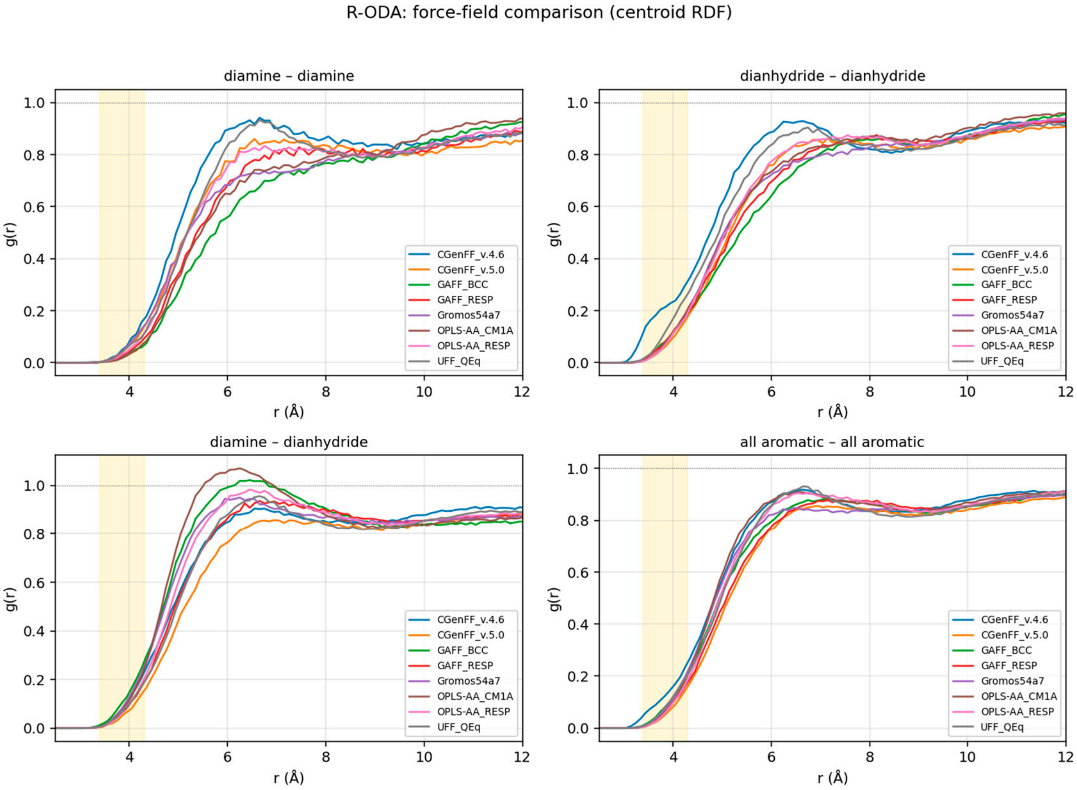

Figure S9. Radial distribution functions (RDFs) of centroid of carbon atoms between the diamine-diamine (top, left), dianhydride-dianhydride (top, right), diamine- dianhydride (bottom, left) and all_aromatic - all_aromatic (bottom, right) of fragment groups of polyimide R-ODA for different all-atom models at T = 1200 K.

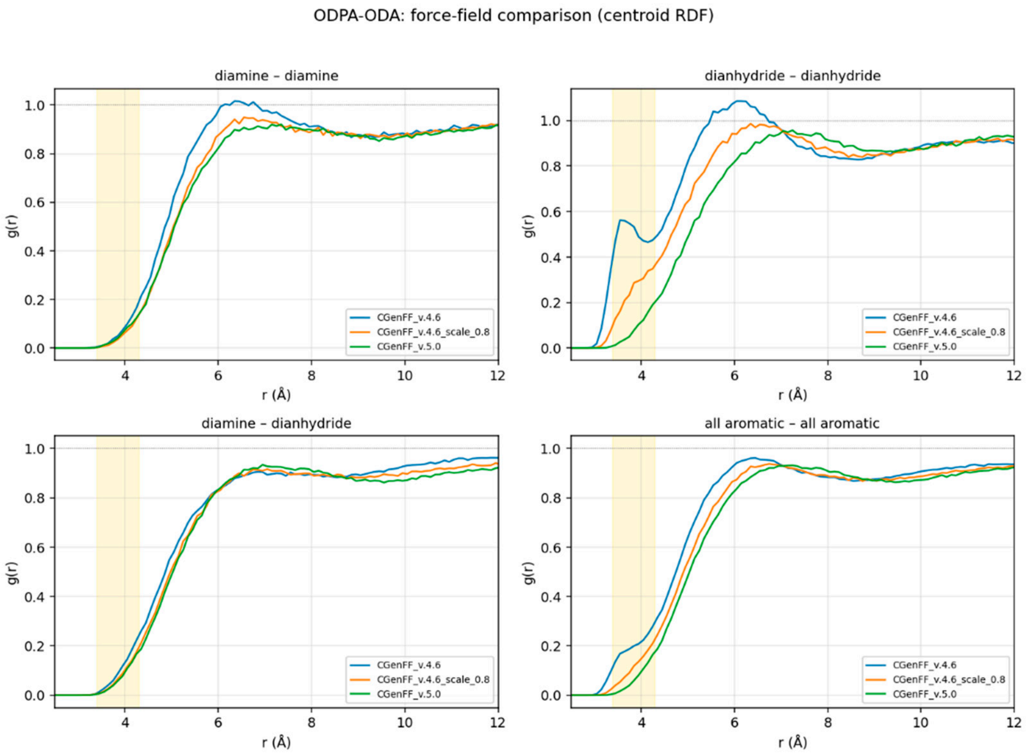

Figure S10. Radial distribution functions (RDFs) of centroid of carbon atoms between the diamine-diamine (top, left), dianhydride-dianhydride (top, right), diamine- dianhydride (bottom, left) and all_aromatic - all_aromatic (bottom, right) of fragment groups of polyimide ODPA-ODA for CGenFF_v.4.6, CGenFF_v.5.0 and CGenFF_v.4.6 with scaled in the 0.8 time partial charges values models at T = 1200 K.

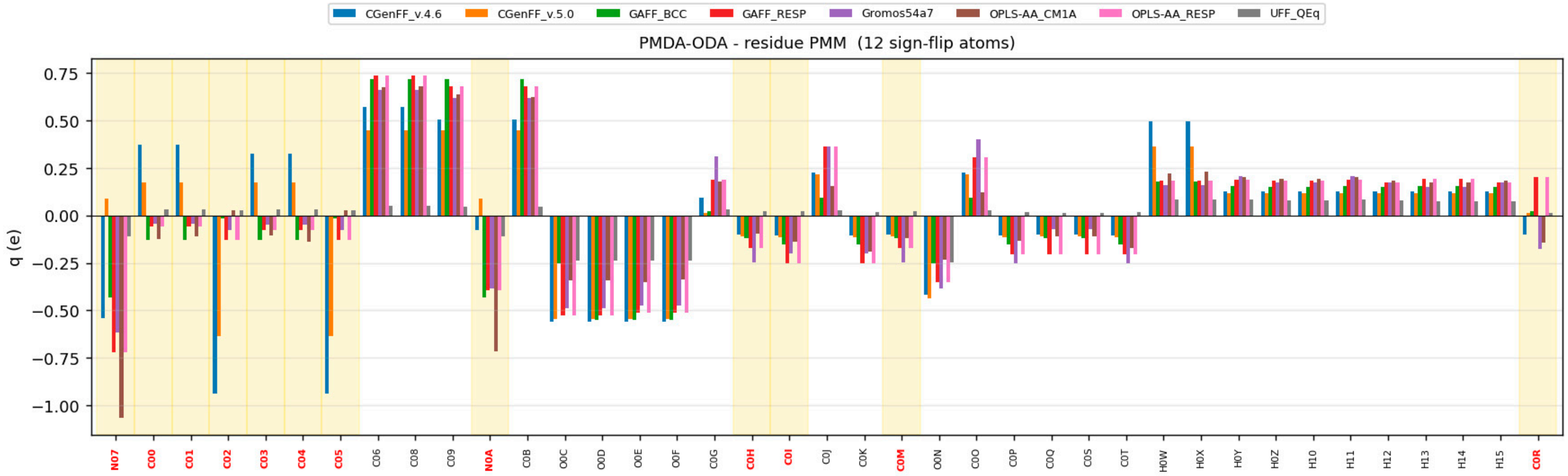
CGenFF_v.4.6
CGenFF_v.5.0
GAFF_BCC
GAFF_RESP
Gromos54a7
OPLS-AA_CM1A
OPLS-AA_RESP
UFF_QEq
PMDA-ODA - residue PMM (12 sign-flip atoms)
q (e)
0.75
0.50
0.25
0.00
−0.25
−0.50
−0.75
−1.00
N07 C00 C01 C02 C03 C04 C05 C06 C08 C09 N0A C0B O0C O0D O0E O0F C0G C0H C0I C0J C0K C0M O0N C0O C0P C0Q C0S C0T H0W H0X H0Y H0Z H10 H11 H12 H13 H14 H15 C0R

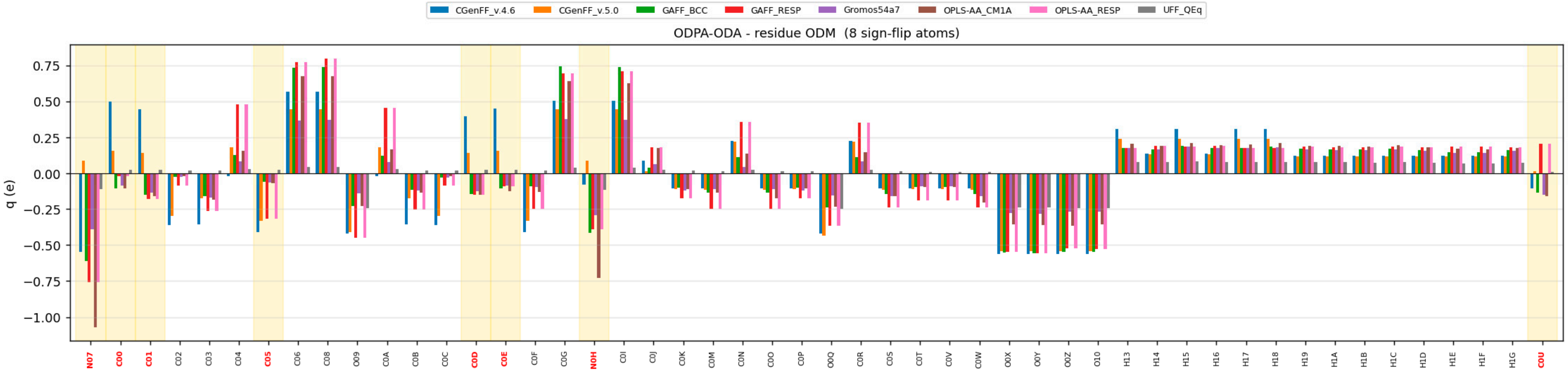
CGenFF_v.4.6
CGenFF_v.5.0
GAFF_BCC
GAFF_RESP
Gromos54a7
OPLS-AA_CM1A
OPLS-AA_RESP
UFF_QEq
ODPA-ODA - residue ODM (8 sign-flip atoms)
q (e)
0.75
0.50
0.25
0.00
−0.25
−0.50
−0.75
−1.00

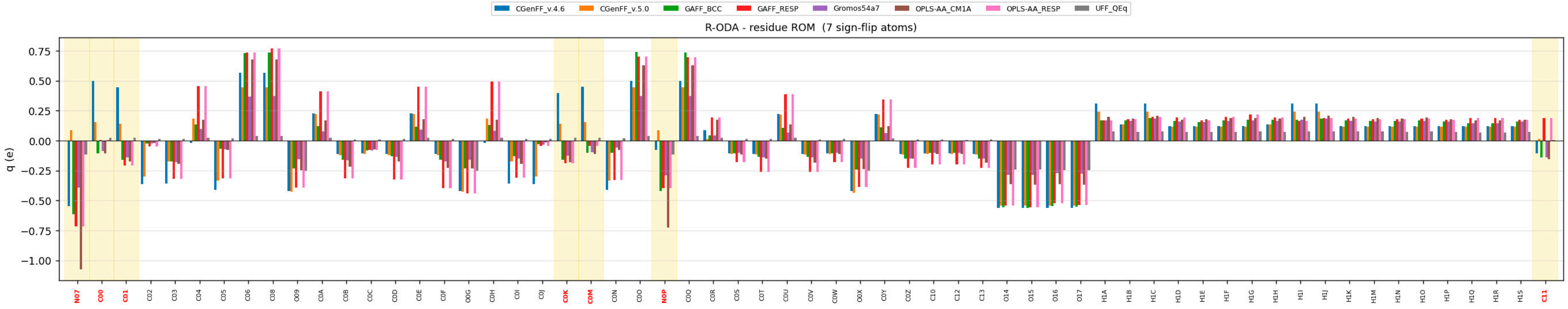

Figure S11. Partial charge values of the middle repeating unit (from second to eleven) of the studied polyimides PMDA-ODA (top), ODPA-ODA (middle), and R-ODA (bottom). The names of the atoms are listed in the repeating units in Figure S12 in the Supplementary Materials.

S3. Thermodynamic flexibility

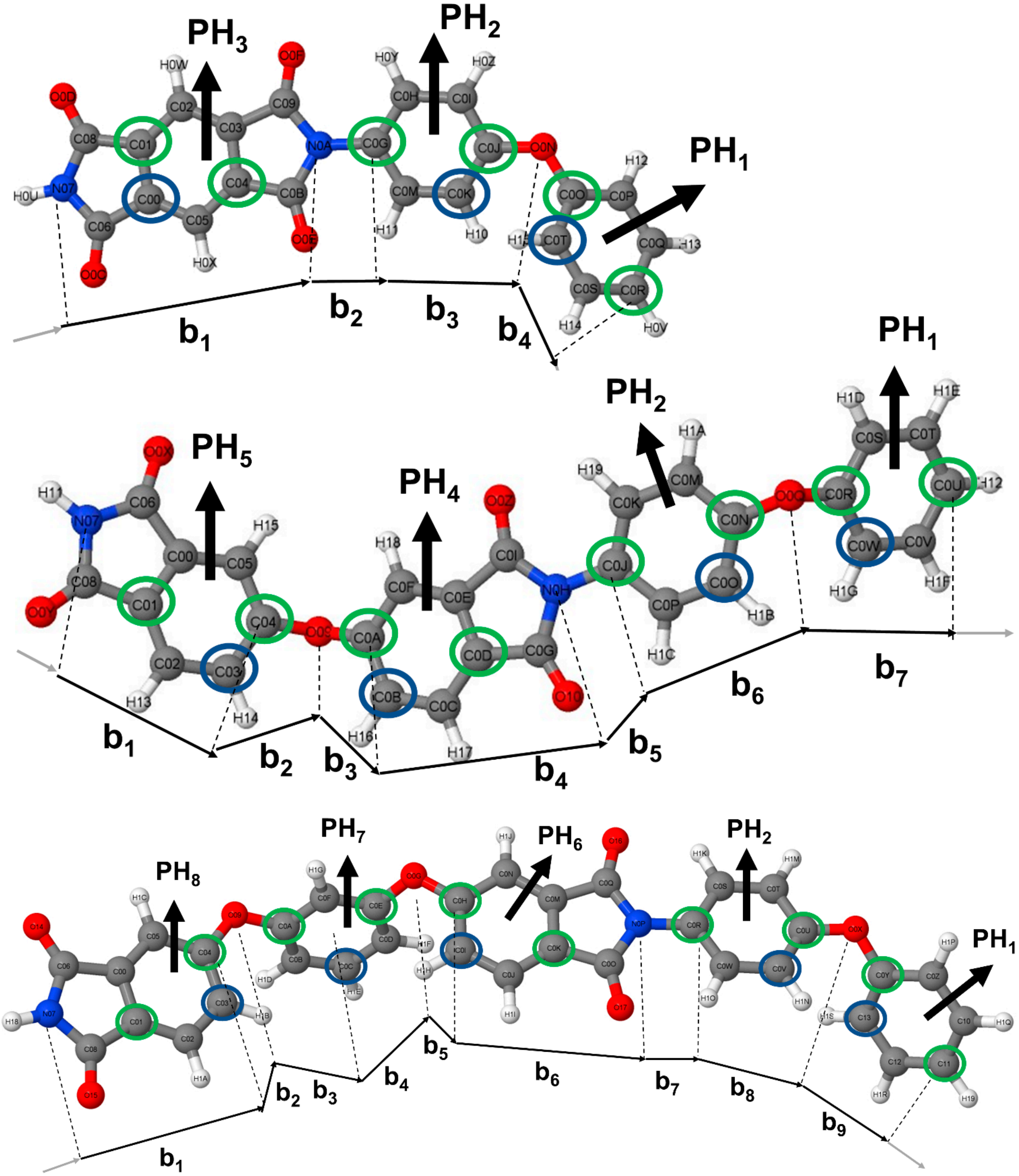

Figure S12. The chemical structure of the repeating unit of PMDA-ODA (top), ODPA-ODA (center) and R-ODA (bottom). The perpendicular arrows indicate the unit normal vectors to the aromatic rings **PH_1-PH_8**. The green and blue circles show the atoms whose coordinates were used to create the vectors in the aromatic rings to calculate the unit normal vectors. The horizontal arrows show the virtual bonds **b_1-b_9** that were used for the calculation of the bond autocorrelation function (BACF) to estimate the value of the persistence length l_p.

The specified set of virtual bond vectors b_i and the correlation function of the first Legendre polynomial were computed:

$$P_1(s)=\frac{\langle b_i\cdot b_{i+s}\rangle}{\langle l\rangle^2}\approx\exp\left(-\frac{L_c(s)}{l_p}\right),$$

(S4)

where ⟨..⟩ denotes an average over configurations, $L_c(s)$ is the contour length of a chain segment of chemical length s measured by summing over the relevant virtual bond lengths, and l_p is the persistence length, which was determined by fitting an exponential function to $P_1(s)$, Figure S13.

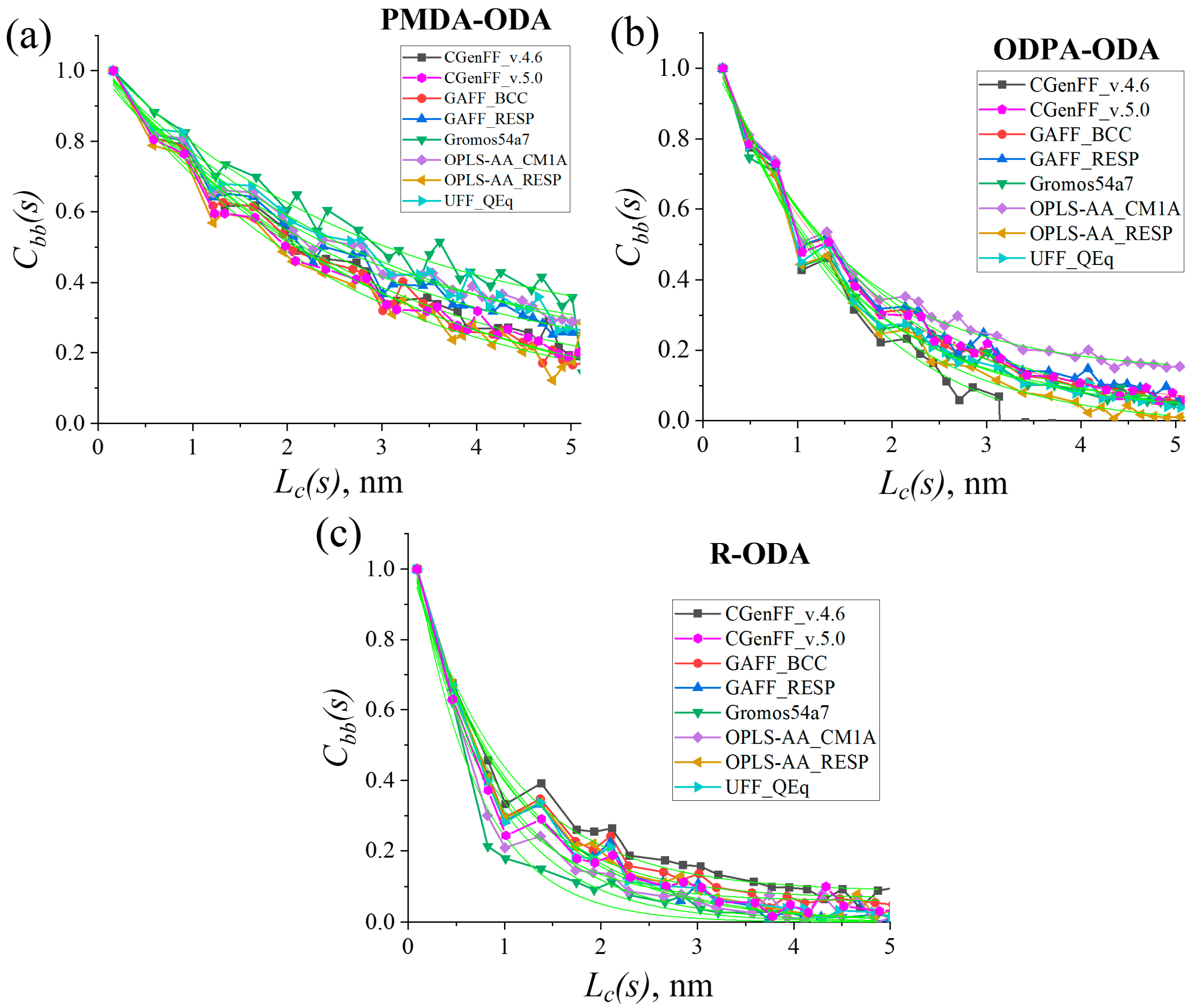

Figure S13. Virtual bond vector autocorrelation functions (BACF) of polyimide (a) PMDA-ODA, (b) ODPA-ODA, and (c) R-ODA. The green lines represent the best fit of BACF with a single exponential function. All simulations were performed at temperature of 1200 K.

Table S4. Persistence length l_p of the studied polyimides obtained via computer simulation.

Structural characteristics	l_p, nm		
Models / Polyimides	PMDA-ODA	ODPA-ODA	R-ODA
CGenFF_v.4.6	2.7±0.3	1.4±0.2	1.0±0.1
CGenFF_v.5.0	3.0±0.1	1.8±0.1	1.0±0.1
GAFF_BCC	3.0±0.4	1.4±0.1	0.9±0.1
GAFF_RESP	2.5±0.3	1.6±0.1	1.1±0.1
Gromos54a7	2.8±0.5	1.7±0.1	0.6±0.1
OPLS-AA_CM1A	2.5±0.3	1.3±0.1	0.8±0.1
OPLS-AA_RESP	2.5±0.3	1.4±0.1	1.1±0.1
UFF_QEq	3.1±0.5	1.6±0.1	1.1±0.1

The value of the Kuhn segment was calculated according S5:

$$b = \frac{\langle H_{e-e}^2 \rangle}{\langle R_{\max} \rangle}, \quad \text{(S5)}$$

where $<H_{e-e}>$is mean square of the chain end-to-end distance and $<R_{max}>$ is the average of the chain contour length, see Figure S3.

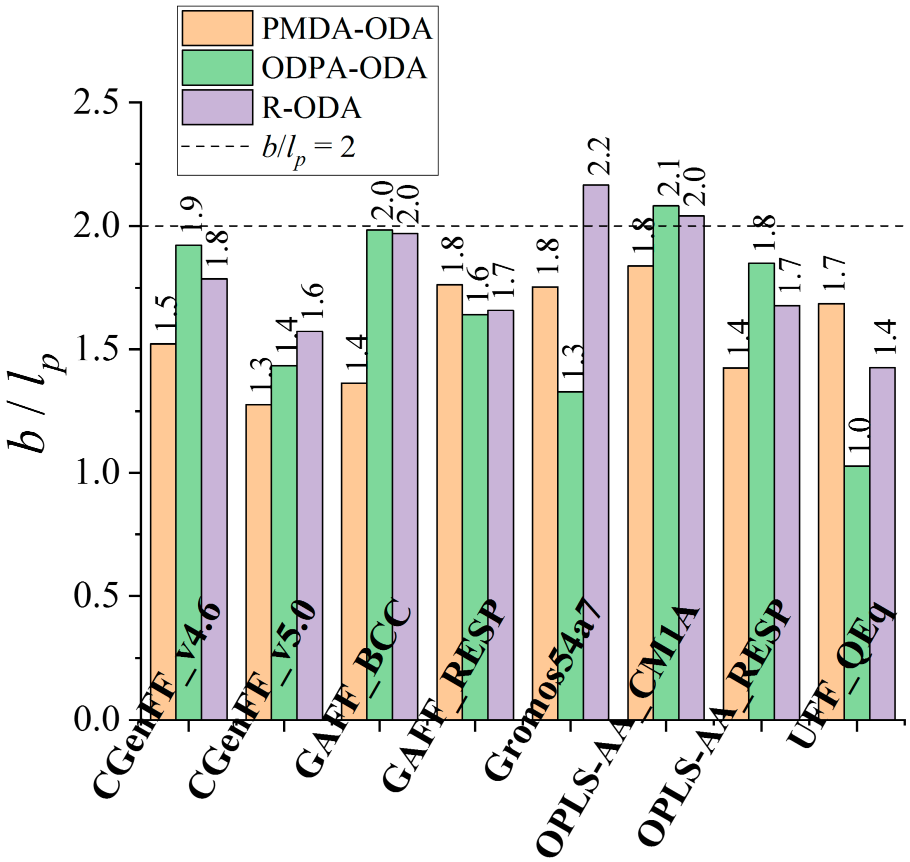

Figure S14. The ratio between the Kuhn segment and the persistence length of the studied PIs for different models. The dashed line shows when the ratio b/l_p is 2.

S4. Local orientational mobility

The first-order Legendre polynomials $P_1(t)$ have been calculated:

$$P_1(t) = \langle \mathbf{b}(0)\mathbf{b}(t) \rangle, \quad \text{(S6)}$$

Where $\mathbf{b}(0)$ and $\mathbf{b}(t)$ are the corresponding normal vectors at the beginning (*t*=0) and at the current moment of time *t*. The angular brackets $\langle \ldots \rangle$ denote the averaging over all 342 rings of 3 independent samples.

$$P_1(t) = \mathrm{A}\exp\left(-(t/\tau)^{\beta}\right), \quad \text{(S7)}$$

where A ≤ 1, τ is the characteristic relaxation time, and *β* is the stretching parameter that considers the nonexponentiality of the relaxation process. The KWW relaxation times *τ* were used to calculate the averaged times τ_c of the rotational relaxation as

$$\tau_c = \frac{\tau_{KWW}}{\beta}\Gamma\left(1/\beta\right), \quad \text{(S8)}$$

where $\Gamma(\ )$ is gamma function.

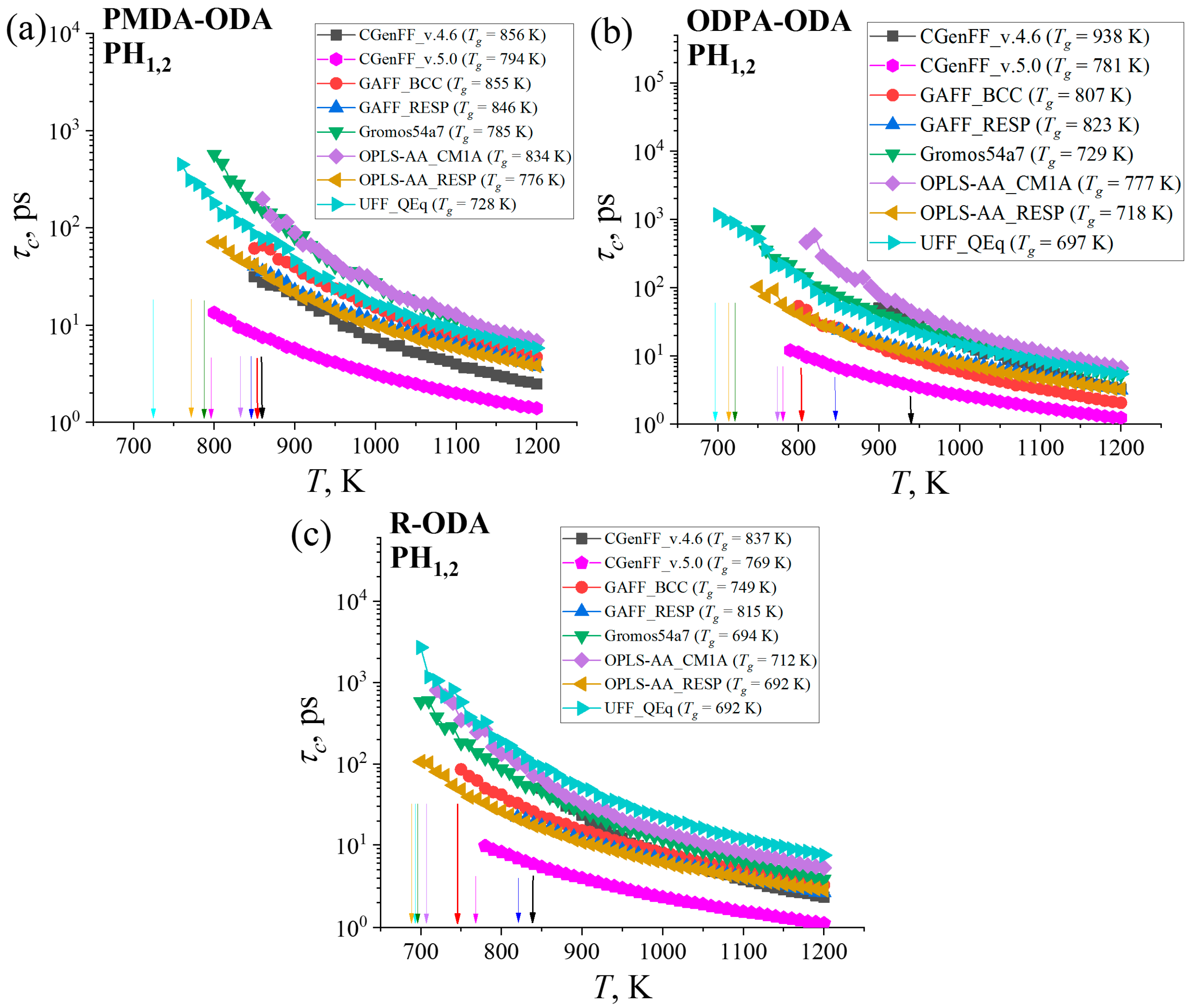

Figure S15. Temperature dependence of the average relaxation times for the orientational mobility of the normal vectors $PH_{1,2}$ for different models of for the PI samples: (a) PMDA-ODA, (b) ODPA-ODA, and (c) R-ODA. The arrows indicate the glass transition temperatures for different models considered.

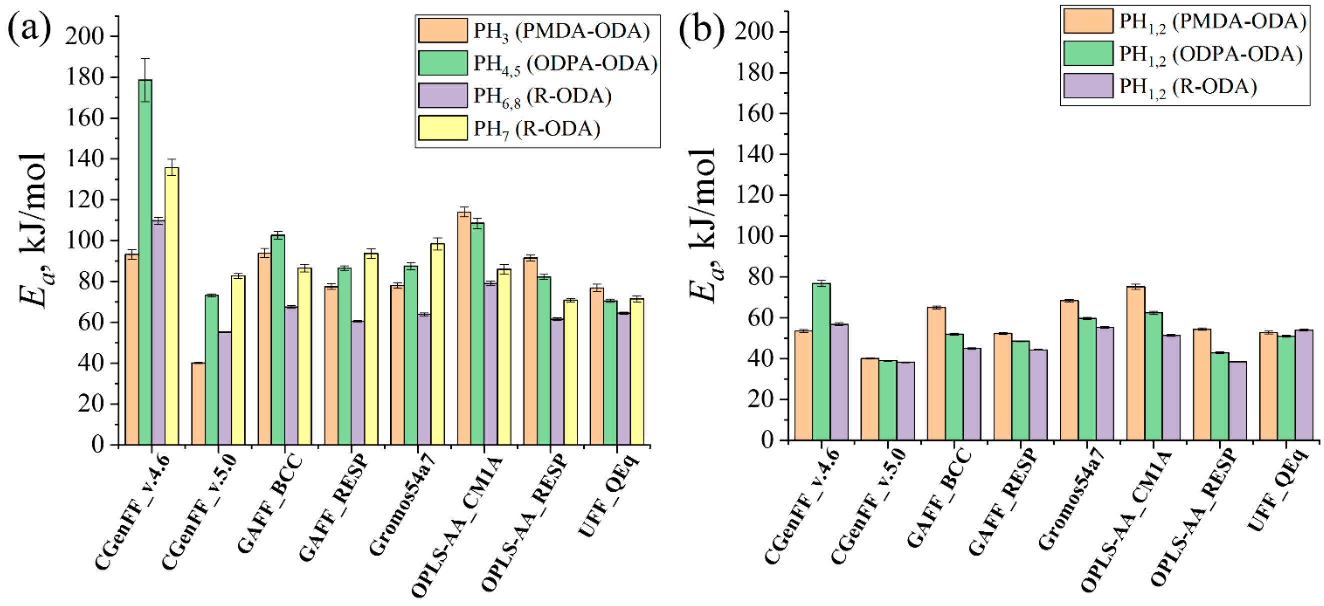

Figure S16. Activation energy values of the normals to the surface of various heterocyclic rings of thermoplastic polyimides located in (a) the dianhydride and (b) the diamine fragment of the repeating unit of the thermoplastic polyimides PMDA-ODA. ODPA-ODA, and R-ODA.

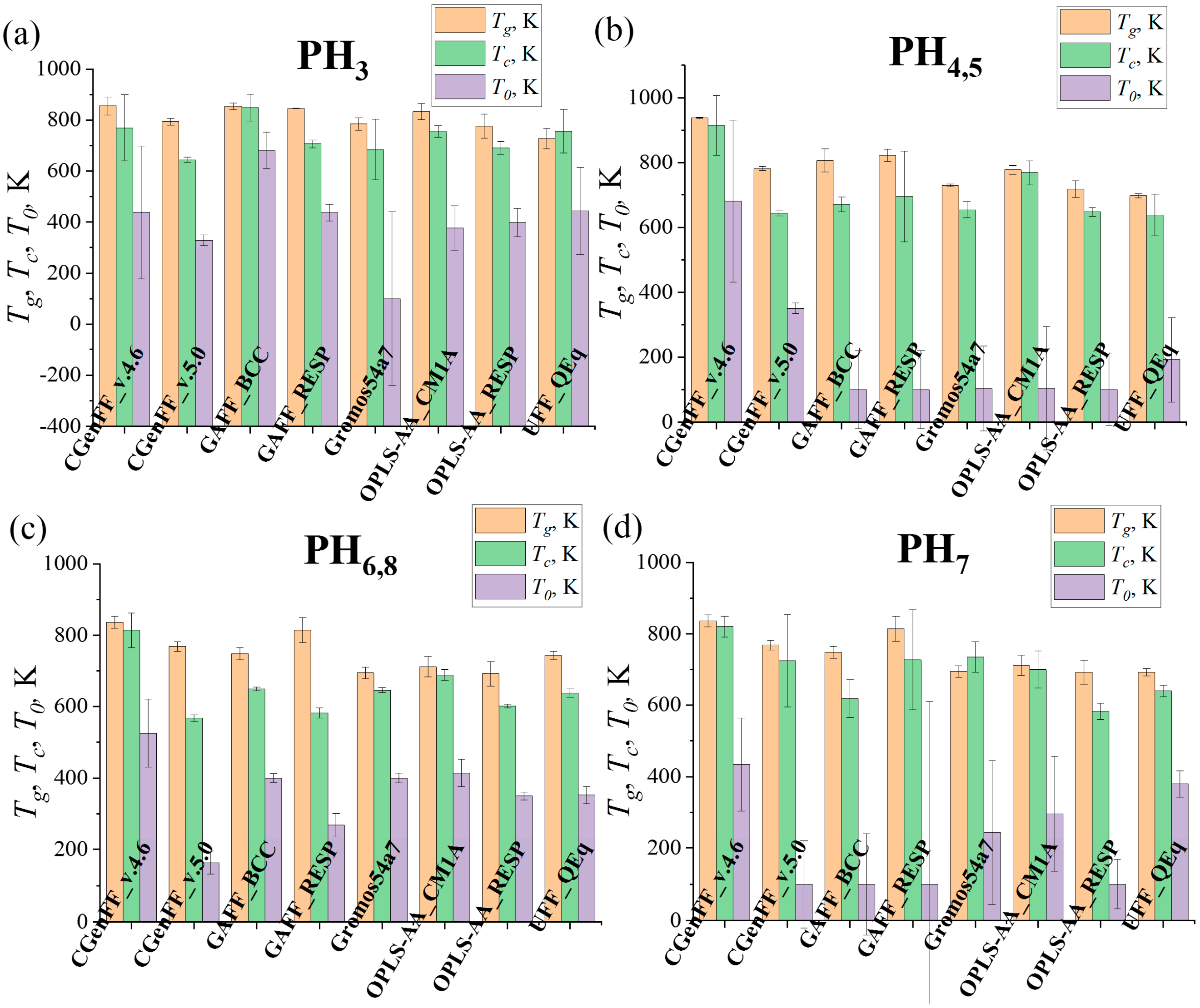

Figure S17. Critical temperatures T_c and T_0 determined using the MCT coupled mode theory and the VFT equation, calculated from the temperature dependencies of the local orientation relaxation time of unit normals to the surface of heterocyclic rings in the dianhydride moiety of the PMDA-ODA repeating unit of polyimides – (a) PH_3, ODPA-ODA – (b) $PH_{4,5}$, and R-ODA – (c) $PH_{6,8}$ and (d) PH_7.

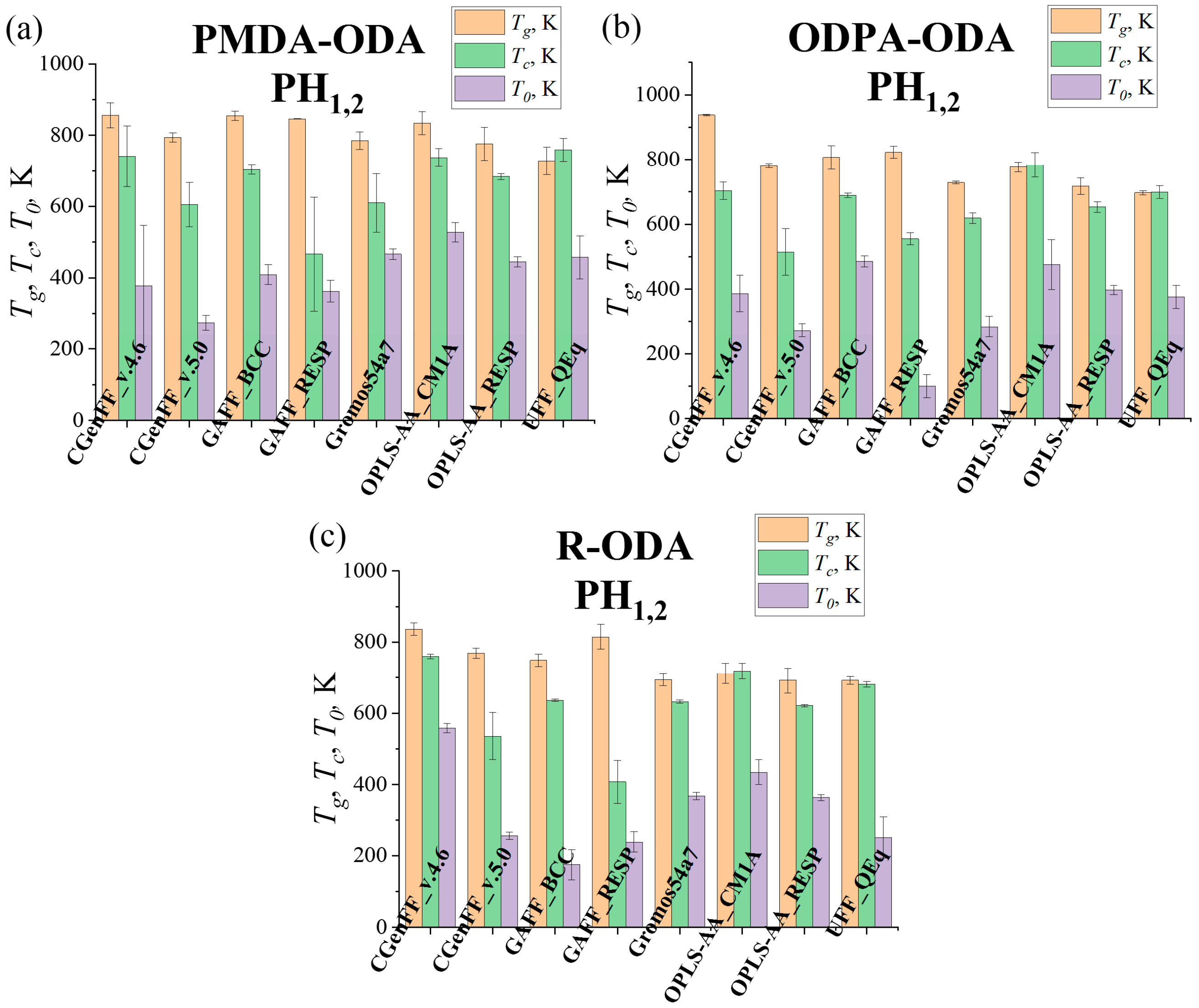

Figure S18. Values of critical temperatures T_c and T_0 determined using the MCT coupled mode theory and the VFT equation, calculated from the temperature dependencies of the local orientation relaxation time of unit normals $PH_{1,2}$ to the surface of heterocyclic rings in the diamine fragment of the repeating unit of polyimides (a) PMDA-ODA, (b) ODPA-ODA, (c) R-ODA.

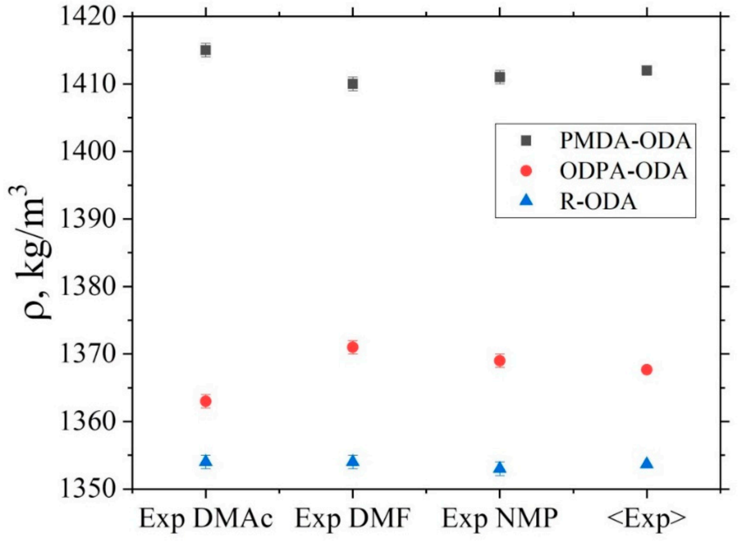

Figure S19. Experimental values of density.

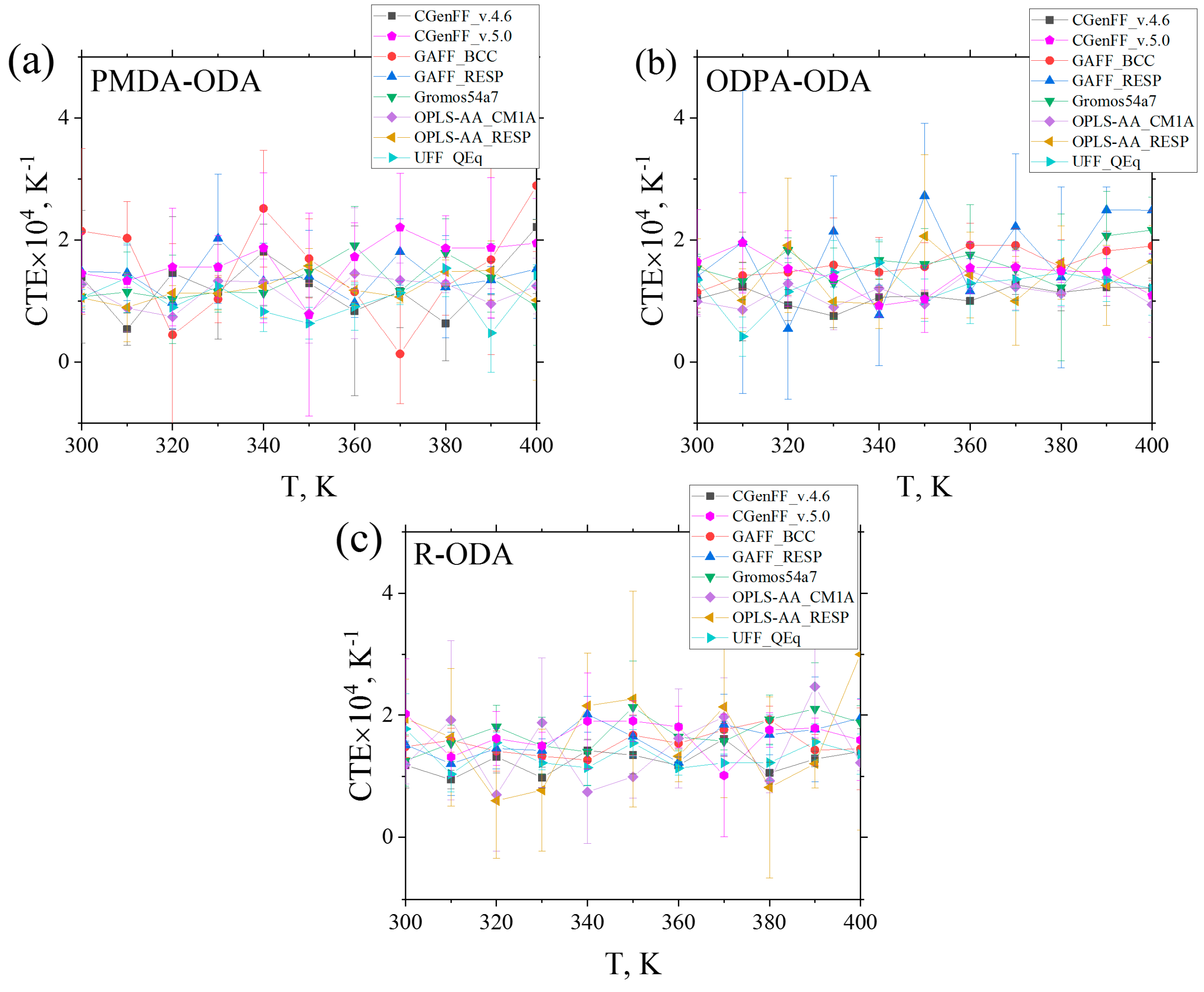

Figure S20. Temperature dependence of the CTE of PIs (a) PMDA-ODA, (b) ODPA-ODA, and (c) R-ODA in different force fields.

Table S5. Average values of CTE for different force fields and experimental values of CTE in the glassy state (in the temperature range of 300-400 K).

	CTE×10⁴, 1/K		
	PMDA-ODA	ODPA-ODA	R-ODA
CGenFF_v.4.6	1.26±0.81	1.09±0.32	1.25±0.29
CGenFF_v.5.0	1.65±0.38	1.42±0.30	1.66±0.29
GAFF_BCC	1.39±0.71	1.62±0.44	1.53±0.30
GAFF_RESP	1.42±0.62	1.62±0.81	1.55±0.40
Gromos54a7	1.29±0.51	1.64±0.58	1.71±0.47
OPLS-AA_CM1A	1.15±0.44	1.13±0.30	1.42±0.87
OPLS-AA_RESP	1.21±0.47	1.41±0.72	1.42±0.29
UFF_QEq	1.05±0.44	1.44±0.35	1.48±0.23
Experiment (DMF)	1.155	1.551	1.677
Experiment (NMP)	1.155	1.536	1.761
Experiment (DMAc)	1.134	1.47	1.716

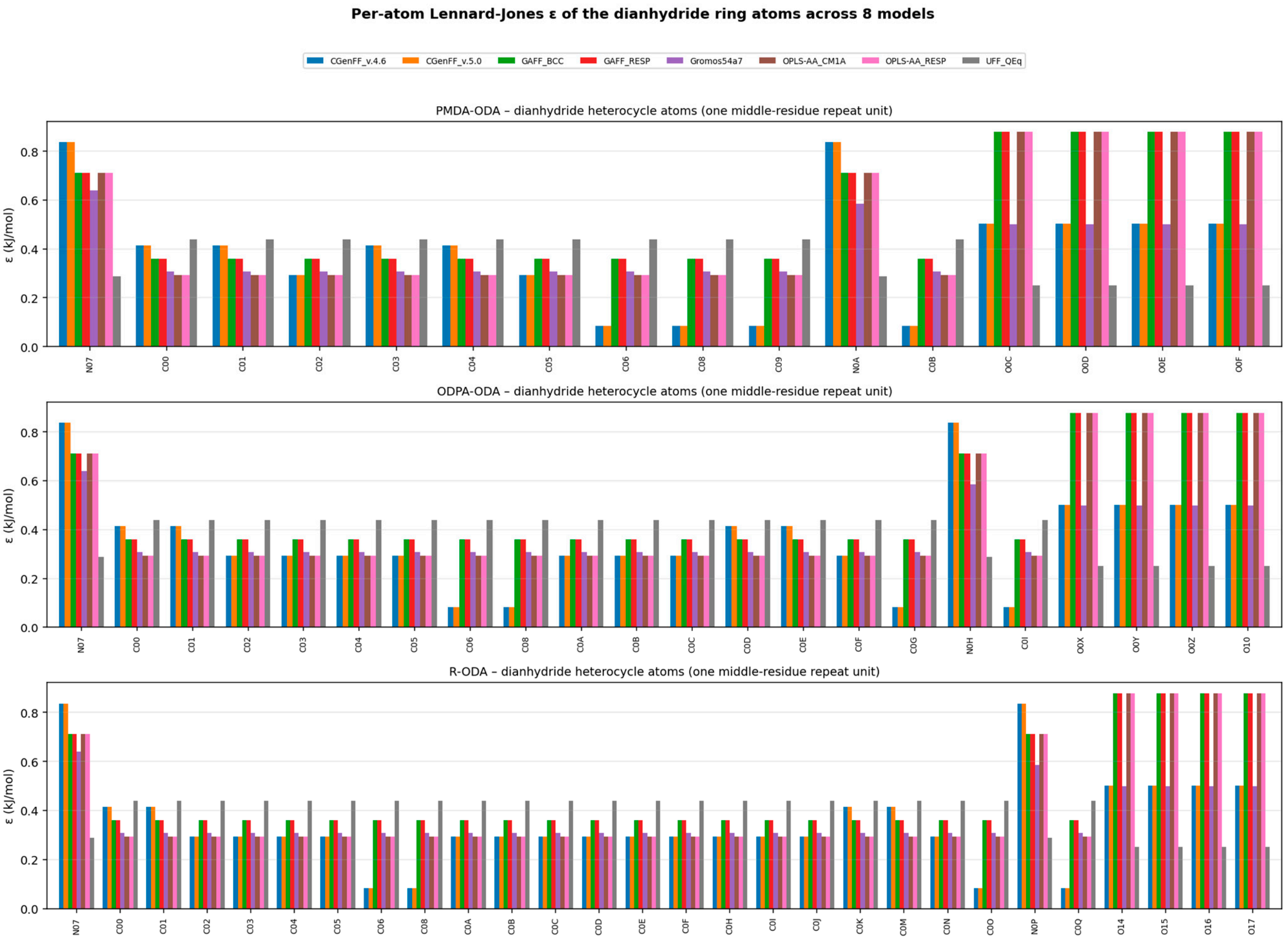

Figure S21. Per-atom Lennard-Jones ε of the dianhydride rings atoms of studied polyimide PMDA-ODA (top), ODPA-ODA (middle), R-ODA (bottom) for different all-atom models. The names of the atoms are listed in the repeating units in Figure S12 in the Supplementary Materials.

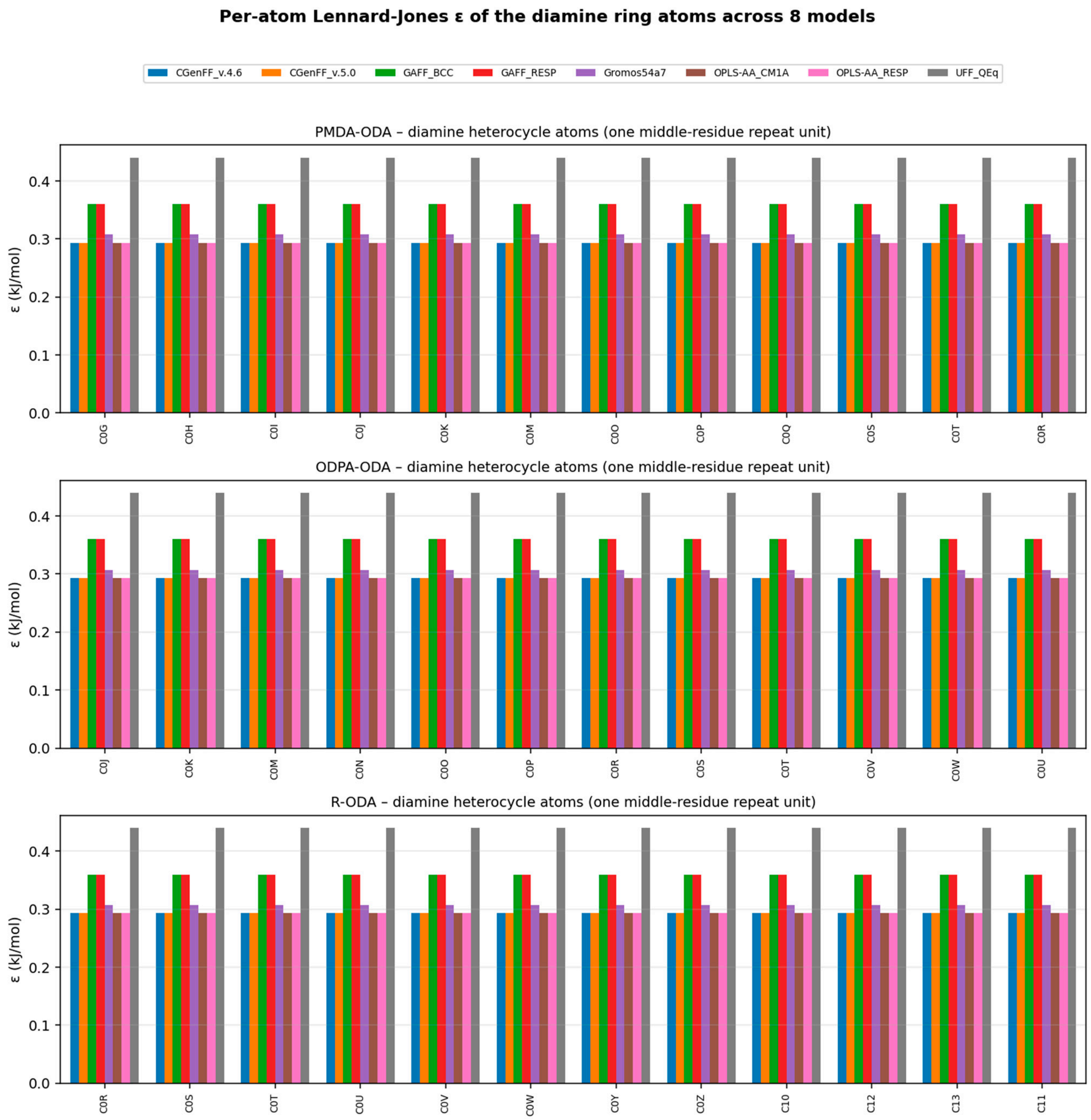

Figure S22. Per-atom Lennard-Jones ε of the diamine rings atoms of studied polyimide PMDA-ODA (top), ODPA-ODA (middle), R-ODA (bottom) for different all-atom models. The names of the atoms are listed in the repeating units in Figure S12 in the Supplementary Materials.

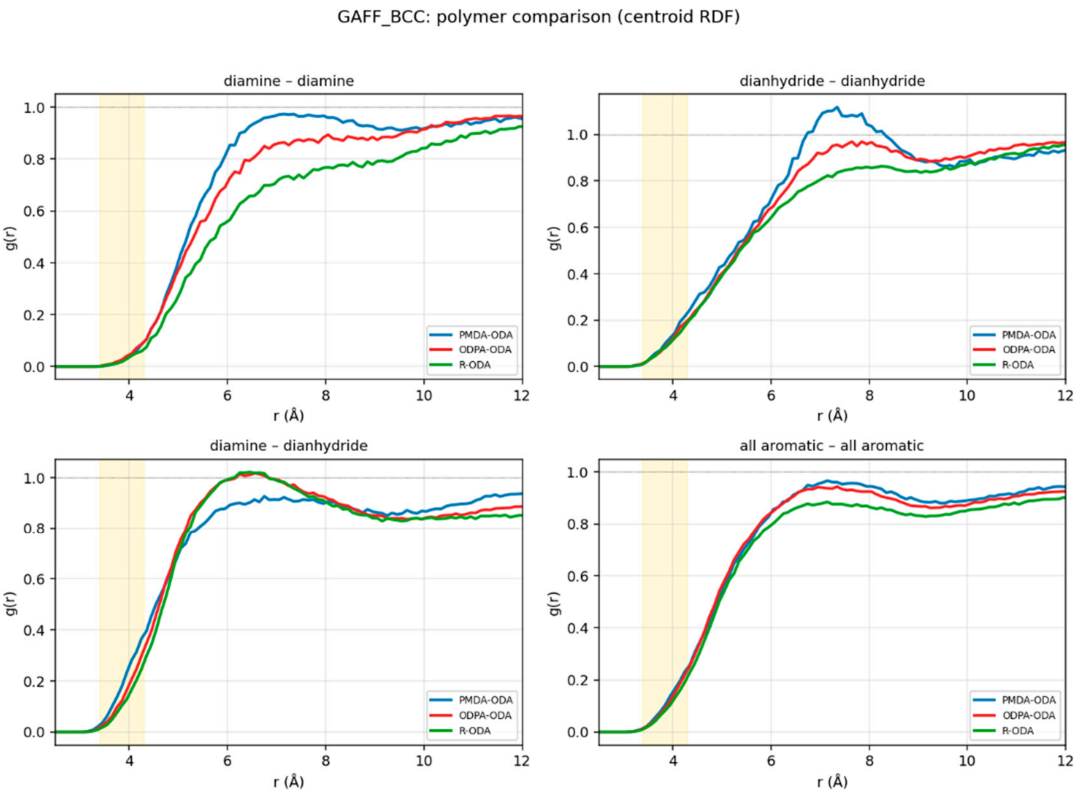

Figure S23. Radial distribution functions (RDFs) of centroid of carbon atoms between the diamine-diamine (top, left), dianhydride-dianhydride (top, right), diamine- dianhydride (bottom, left) and all_aromatic - all_aromatic (bottom, right) of fragment groups of considered polyimides PMDA-ODA, ODPA-ODA and R-ODA for GAFF_BCC model at T = 1200 K.

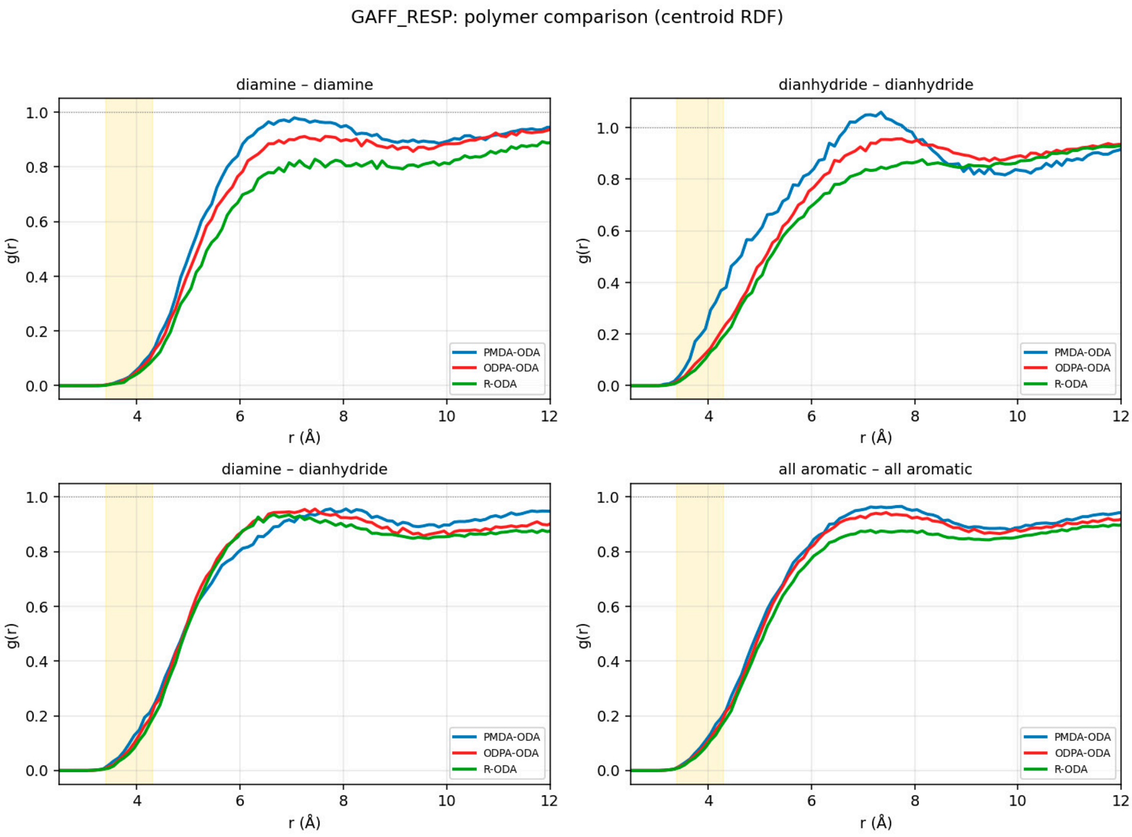

Figure S24. Radial distribution functions (RDFs) of centroid of carbon atoms between the diamine-diamine (top, left), dianhydride-dianhydride (top, right), diamine- dianhydride (bottom, left) and all_aromatic - all_aromatic (bottom, right) of fragment groups of considered polyimides PMDA-ODA, ODPA-ODA and R-ODA for GAFF_RESP model at T = 1200 K.

S5. Mechanical Properties

The uniaxial deformation procedure involves an affine change in atomic coordinates with a constant deformation rate along one direction of the coordinate axes (*X*, *Y*, or *Z*). The values of the pressure tensor P_i, where i = x, y, z, and the dimensions of the simulation box L_i in the deformation direction were increased. The mechanical properties(the values of σ and ε)were evaluated as follows [7]:

$$\sigma = -P_i, \quad \text{(S9)}$$

$$\varepsilon = \frac{L_i - L_{0i}}{L_{0i}}, \quad \text{(S10)}$$

where L_{0i} is simulation cell edge size in the deformation direction at $t = 0$.

The initial section of σ(ε) (up to 2% strain) is the linear viscoelastic regime and can be approximated by the linear dependence $\sigma = E\varepsilon$ (S11), where *E* is Young's modulus. It should be emphasized that, in some cases, the specimen had an initial residual stress, and the dependence σ(ε) did not always go beyond zero. To calculate the value of *E* in this case, the dependence of σ(ε) was shifted to zero values of σ and ε. The errors in the calculations of the elastic modulus were evaluated as standard deviations from the average value of *E* obtained by averaging over three independent configurations and three strain directions, i = x, y, z. The yield strength σ_y (the onset of plastic deformation in the material) was calculated using a transformed stress-strain relationship: σ(ε) was converted from the engineering strain function (ε=λ-1) to the relationship $\varepsilon_t = (\lambda^2 - \lambda^{-1})$, where $\lambda = L_i / L_{i0}$. For this purpose, the obtained curve was approximated by the dependence [8].

$$\sigma = \sigma_y + G_h(\lambda^2 - \lambda^{-1}), \quad \text{(S12)}$$

where G_h is strain-hardening modulus (slope of the linear dependence σ(ε) in the deformation regime after yielding). The approximation region ε_t was chosen from 0.75 to 2.25.

Table S6. Mechanical characteristics (Young's modulus E and yield strength σ_y) of the studied polyimides obtained via computer simulations. The error bars were calculated as the standard deviation of the samples, which consisted of three independent configurations and three deformation directions.

Mechanical Characteristics	E, GPA			σ_y, MPa		
Models / Polyimides	PMDA-ODA	ODPA-ODA	R-ODA	PMDA-ODA	ODPA-ODA	R-ODA
CGenFF_v4.6	3.45±0.25	4.47±0.13	3.99±0.26	203±34	264±5	233±13
CGenFF_v5.0	3.69±0.1	3.43±0.2	3.38±0.2	196±22	168±13	159±10
GAFF_BCC	2.99±0.37	3.2±0.3	3.2±0.2	190±34	213±42	194±26
GAFF_RESP	3.01±0.14	2.6±0.5	2.89±0.15	172±19	143±13	177±22
Gromos54a7	3.69±0.34	2.9±0.3	3.23±0.07	253±98	191±8	193±4
OPLS-AA_CM1A	3.96±0.27	3.98±0.17	4.06±0.21	292±15	291±10	250±21
OPLS-AA_RESP	3.6±0.1	3.5±0.1	3.37±0.09	233±36	210±10	197±17
UFF_QEq	3.8±0.3	2.0±0.4	3.1±0.6	237±17	180±38	187±44

Table S7. Mechanical characteristics (Strain-hardening Moduli, G_h) of the studied polyimides obtained by computer simulations.

Mechanical Characteristics	G_h, MPa		
Models / Polyimides	PMDA-ODA	ODPA-ODA	R-ODA
CGenFF_v.4.6	114±6	112±6	48±2
CGenFF_v.5.0	80±5	63±4	50±2
GAFF_BCC	78±4	59±3	37±2
GAFF_RESP	96±5	74±4	34±2
Gromos54a7	89±4	55±3	38±2
OPLS-AA_CM1A	114±6	63±3	41±2
OPLS-AA_RESP	78±4	65±4	49±2
UFF_QEq	69±3	56±3	33±4

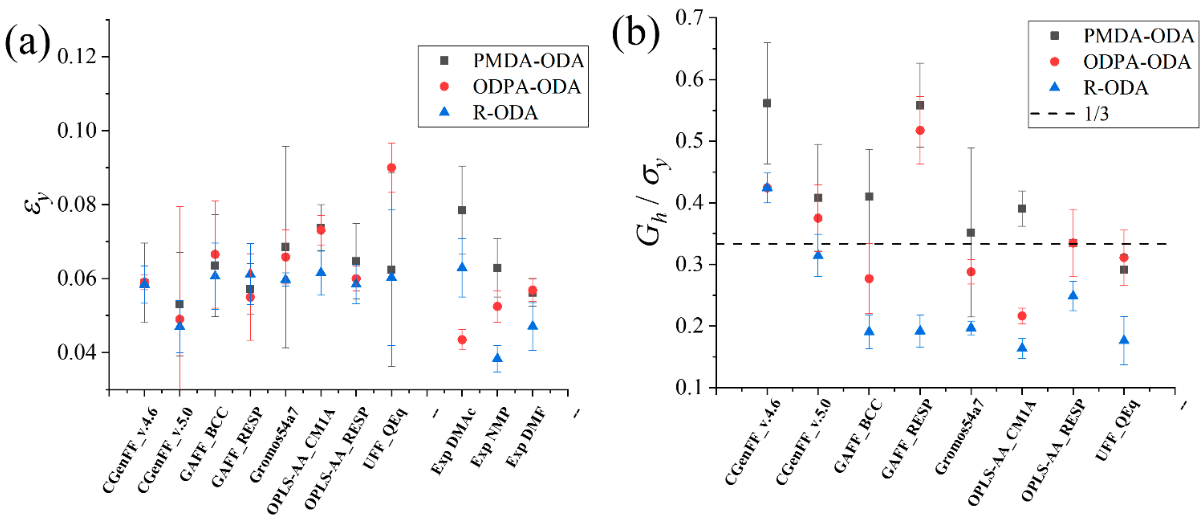

Figure S25. The mechanical characteristics of studied polyimide calculated from the values of Young's modulus E, yield strength σ_y and strain-hardening modulus G_h: (a) elastic yield strain $\varepsilon_y = \sigma_y/E$ and (b) Considere's criterion G_h/σ_y, the dashed line corresponds to Considere's criterion that determines the strength performance of the material.

.

References

[1] GROMACS 2022.6 Manual, 2022. https://manual.gromacs.org/documentation/2022-current/manual-2022.6.pdf.

[2] P.N. Patrone, A. Dienstfrey, A.R. Browning, S. Tucker, S. Christensen, Uncertainty quantification in molecular dynamics studies of the glass transition temperature, Polymer. 87 (2016) 246–259. doi:10.1016/j.polymer.2016.01.074.

[3] F.J. Carmona Esteva, Y. Zhang, E.J. Maginn, Y.J. Colón, Consistent and reproducible computation of the glass transition temperature from molecular dynamics simulations, The Journal of Chemical Physics. 161 (2024). doi:10.1063/5.0207835.

[4] Q. Yang, X. Chen, Z. He, F. Lan, H. Liu, The glass transition temperature measurements of polyethylene: determined by using molecular dynamic method, RSC Advances. 6 (2016) 12053–12060. doi:10.1039/C5RA21115H.

[5] V.M. Nazarychev, A.A. Pavlov, A.M. Kamalov, M.E. Borisova, A.L. Didenko, E.M. Ivan'kova, V.E. Kraft, G. V Vaganov, A.L. Nikolaeva, A.S. Ivanova, V.K. Lavrentiev, E.N. Popova, I. V Abalov, A.N. Blokhin, A.N. Bugrov, V. V Kudryavtsev, The Effect of Synthesis Conditions and Chemical Structure of Thermoplastic Polyimides on Their Thermomechanical Properties and Short-Term Electrical Strength, Polymers. 17 (2025) 1385. doi:10.3390/polym17101385.

[6] K. Hackenstrass, N. Tabudlong Jonasson, M. Hartwig-Nair, T. Rosén, S. Florisson, M. Wohlert, Analysing π–π-stacking interactions in lignin nanoparticles from molecular simulations – insights and lessons learned, Faraday Discussions. 263 (2026) 52–64. doi:10.1039/D5FD00052A.

[7] V.M. Nazarychev, A. V. Lyulin, S. V. Larin, A.A. Gurtovenko, J.M. Kenny, S. V. Lyulin, Molecular dynamics simulations of uniaxial deformation of thermoplastic polyimides, Soft Matter. 12 (2016) 3972–3981. doi:10.1039/C6SM00230G.

[8] V.M. Nazarychev, A.Y. Dobrovskiy, S. V. Larin, A. V. Lyulin, S. V. Lyulin, Simulating local mobility and mechanical properties of thermostable polyimides with different dianhydride fragments, Journal of Polymer Science Part B: Polymer Physics. 56 (2018) 375–382. doi:10.1002/polb.24550.
